# Glacier Melt as a Source of Mercury: Implications for Ecosystem Recovery and Environmental Trends

**DOI:** 10.1021/acs.est.5c16308

**Published:** 2026-04-15

**Authors:** Davide Mattio, Stéphane Guédron, Pierre Sabatier, Yann Bertrand, Nicolas Bonfanti, Sylvain Campillo, Aurélien Dommergue, David Gateuille, Elsa Gautier, Antonio Martínez Cortizas, Emmanuel Naffrechoux, Antoine Rabatel, Hélène Angot

**Affiliations:** † 57507 Univ. Grenoble Alpes, CNRS, INRAE, IRD, Grenoble INP, IGE, Grenoble 38400, France; ‡ ISTerre, 27056 Univ. Grenoble Alpes, Université Savoie Mont Blanc, CNRS, IRD, UGE, Grenoble 38610, France; § EDYTEM, CNRS, Université Savoie Mont Blanc, Le Bourget du Lac 73376, France; ∥ Université Savoie Mont Blanc, INRAE, CARRTEL, Thonon-Les-Bains 74200, France; ⊥ EcoPast (GI-1553), Facultade de Bioloxía, Universidade de Santiago de Compostela, Santiago de Compostela 15782, Spain

**Keywords:** mercury, glacier melt, alpine lakes, sediment accumulation rates, atmospheric deposition, climate change, ecosystem recovery

## Abstract

Mercury (Hg) atmospheric emissions in Europe peaked in the mid-20th century and have since declined due to environmental policies, reducing atmospheric deposition to ecosystems. However, regional disparities have emerged as climate change can remobilize Hg reservoirs, complicating efforts to assess environmental recovery. This study investigates Hg accumulation rates in lacustrine sediments from two neighboring high-altitude alpine lakes in the French Alps: Grand Lake (GDL), a rain-fed lake reflecting regional atmospheric deposition, and Eychauda Lake (EYC), a proglacial lake supplied by glacial meltwater and derived sediment. Sediment chronologies for both lakes were established using short-lived radionuclides, allowing for the reconstruction of Hg deposition over the past century. Comparison of the Hg records from both lakes revealed contrasting trends: GDL responds to regional anthropogenic emissions, while EYC shows a still increasing Hg accumulation rate over recent decades. This discrepancy and its correlation with erosion proxies suggest that glacier melting has released Hg stored in the ice, masking the expected trend of reduced regional emissions. These findings illustrate the importance of accounting for climate-mediated remobilization of Hg when evaluating the effectiveness of environmental policies as this process can impose a climate change-driven penalty that delays ecosystem recovery.

## Graphical abstract

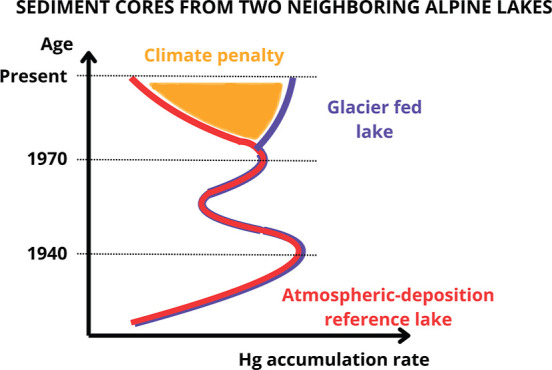

## Introduction

Mercury (Hg) is a global pollutant with serious consequences for both the ecosystem and human health.^1^ Its atmospheric transport as gaseous elemental Hg, Hg(0), allows it to travel long distances before being deposited into aquatic and terrestrial ecosystems through both wet and dry deposition.^2^ Once deposited, Hg can be converted into methylmercury (MeHg), a highly neurotoxic compound that bioaccumulates in aquatic food webs, increasing exposure risks for wildlife and humans, primarily through fish consumption.^1^ International agreements like the Minamata Convention on Mercury seek to curb anthropogenic Hg emissions to reduce global Hg pollution.^3^ However, despite global efforts to reduce emissions, Hg persists in the environment, cycling between atmospheric, terrestrial, and aquatic reservoirs over time scales ranging from centuries to millennia.^4^

The Minamata Convention on Mercury, ratified in 2017, requires regular evaluations of its effectiveness based on available environmental and scientific data such as Hg concentrations in air, water, and biota (Article 22). A key aspect of these evaluations is assessing whether policy interventions can be linked to observed changes in Hg levels. However, climate change adds another layer of complexity to this process.^5^

One major challenge in Hg mitigation is the presence of secondary sources of Hg, i.e., previously deposited Hg that can be remobilized into active biogeochemical cycles.^6–8^ In this paper, we adopt the definition proposed by Dastoor et al. (2025),^9^ which distinguishes secondary emissions (from both contemporary and historical sources, natural or anthropogenic) from legacy emissions—re-emissions originating exclusively from primary emissions that occurred at least one year prior, without implying source attribution. These recycling processes can undermine the effectiveness of current emission reduction policies.^7,10^

While several studies have explored Hg remobilization from sources such as oceans, permafrost, and soils,^11–13^ relatively little attention has been given to Hg secondary emissions from glacial melt beyond the Greenland ice sheet^14–16^ and the Tibetan Plateau.^17–20^ Outside of the polar areas, alpine regions are greatly affected by climate-induced glacier retreat^21–23^ and can thus act as secondary sources of pollutants previously trapped in ice or stored in the surrounding catchment.^24^ While this process is well-documented for persistent organic pollutants (POPs), particularly in the European Alps,^25–28^ the remobilization of Hg remains poorly documented.^29^

Alpine lake sediments serve as natural archives for reconstructing historical and recent Hg deposition trends and provide insight into climate-driven disturbances.^30,31^ In glacierized watersheds, Hg inputs to lake sediments originate from a complex variety of sources and processes, which include (1) dry and wet atmospheric deposition, (2) the release of Hg stored in glacial ice, and to a lesser extent, in dissolved meltwater content fractions,^32,33^ (3) input of Hg­(II) associated with leached organic matter (OM) or terrigenous particles from the catchment,^34^ and (4) in-lake biological uptake (i.e., of Hg­(II) and Hg(0)).^35,36^ (Figure 1) This study analyzes sediment cores from two nearby alpine lakes, one influenced by glacier melt and the other not, to evaluate how glacier retreat affects mercury accumulation in lake sediments. Undisturbed sediment cores with a robust chronological framework were analyzed for Hg concentrations along with organic and inorganic elemental and isotopic tracers to identify Hg sources and carrier phases within the catchment. These data were further used to assess how glacial retreat influences sedimentation rates, lake productivity, and catchment erosion, thereby affecting Hg delivery to the lake. Finally, the records were compared with regional Hg and glacier reconstructions to isolate and quantify the role of climate change in remobilizing Hg, thereby providing a direct estimate of the climate change-driven penalty affecting policy assessments.

**Figure 1:**
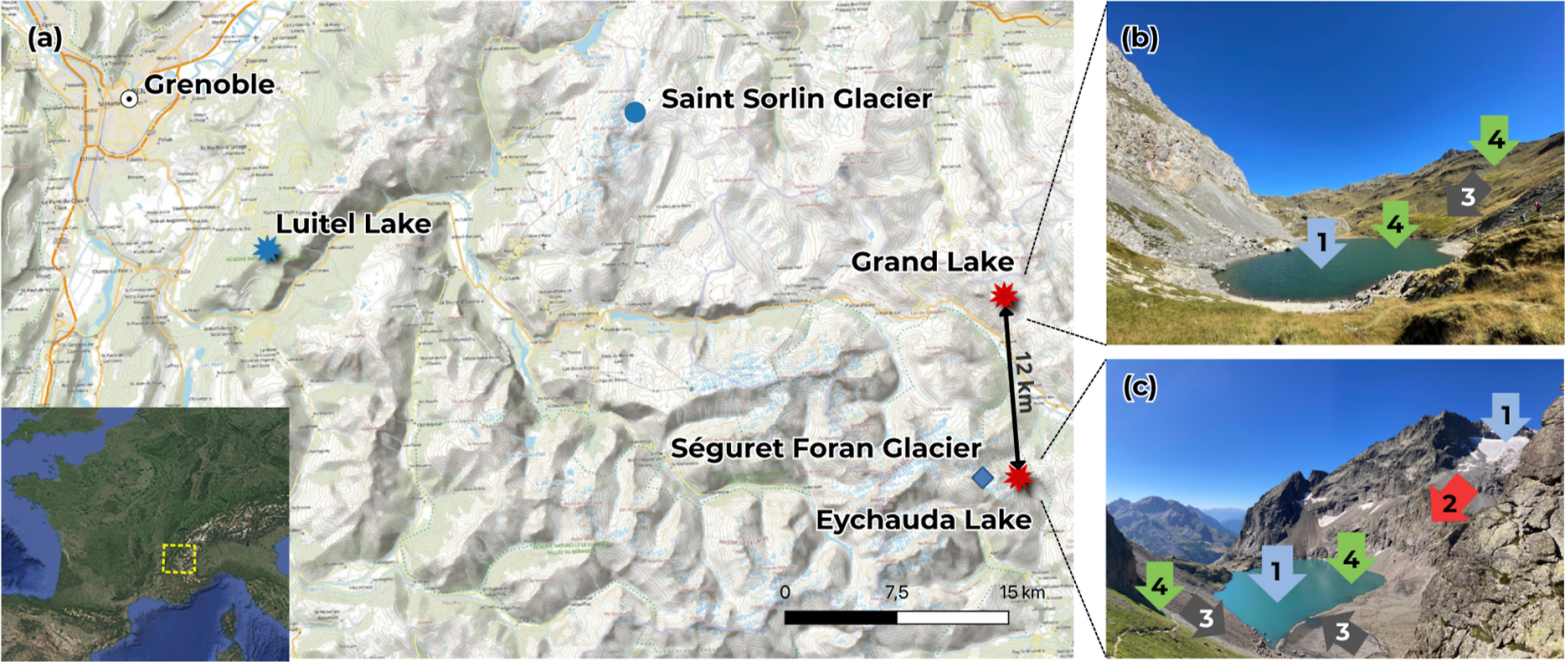
(a) Map of the study area showing the locations of the investigated lakes and nearby glaciers referenced in the text. On the right, photographs of the two study lakes: (b) Grand Lake (nonglacier-fed) and (c) Eychauda Lake (glacier-fed). Arrows indicate the main potential sources and pathways of mercury (Hg) to the lakes: (1) atmospheric deposition, (2) release of secondary Hg from glacial melt, (3) catchment erosion, and (4) Hg uptake and cycling through the biological pump. These processes are considered when interpreting the differences in sediment Hg accumulation between the glacier-fed and nonglacier-fed lakes.

## Material and Methods

### Study Sites

#### Nonglacier-Fed Lake: Grand Lake (GDL)

Grand Lake is situated at an elevation of 2282 m above sea level (m a.s.l.) and spans approximately 0.047 km^2^ (Table S5). It is surrounded by landscape with gentle slopes and drains a small, confined catchment of 3.32 km^2^.^37^ Its high-altitude position and limited hydrological connectivity suggest that Grand Lake functions primarily as a “rain gauge” lake —i.e., a system where atmospheric deposition is the dominant source of input, with limited influence from surface runoff. Although a debris cone is present on the western slope and a small delta is located at the northern edge, indicating some degree of catchment contribution,^38^ the lake’s morphology and limited inflows support its classification as a reliable archive for reconstructing atmospheric Hg deposition. This is consistent with previous studies showing that high-elevation lakes with isolated catchments provide robust records of atmospheric deposition.^39,40^

#### Glacier-Fed Lake: Eychauda Lake (EYC)

Eychauda Lake is located at an elevation of 2517 m a.s.l., with a surface area of 0.151 km^2^ and a watershed area of 2.7 km^2^.^41^ This proglacial lake lies just a few hundred meters downstream of the Séguret Foran Glacier, which feeds it with meltwater (Figure S2). At the northern end of the lake, the glacial stream forms a delta marked by a frontal moraine.^41^ Mass-balance data for the Séguret Foran Glacier^42^ indicate a pronounced negative trend beginning in the 1980s (Figure 4d). This pattern is consistent with observations from neighboring glaciers such as the Saint-Sorlin Glacier (SOR; see Figure 1) and with reports for most glaciers in the Alps.^21^

**Figure 2:**
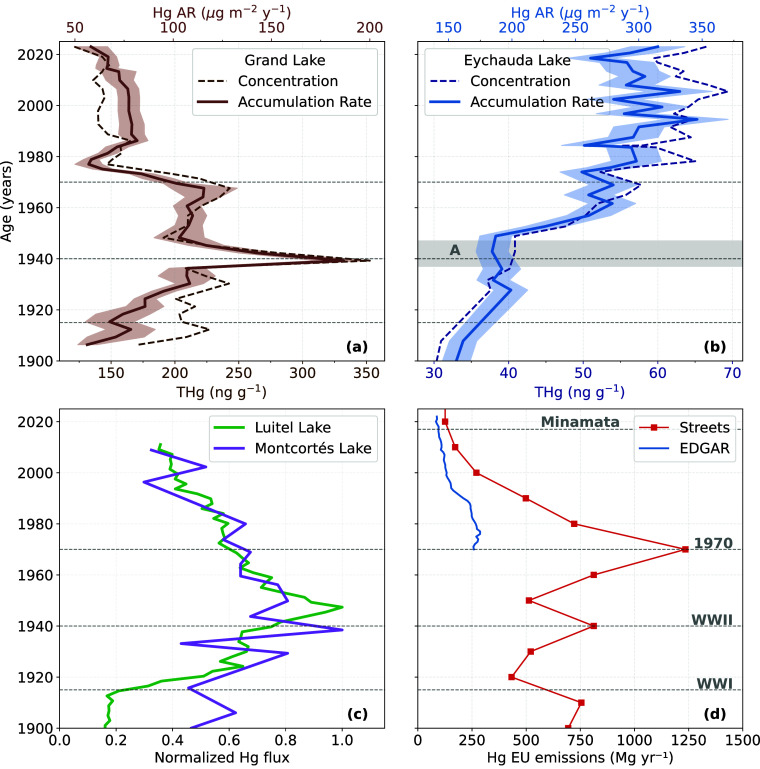
(a,b) Hg accumulation rate (AR) and concentration profiles for the nonglacier-fed lake (GDL, brown) and the glacial-fed lake (EYC, blue). Shaded areas represent the uncertainty associated with accumulation rates. Period A corresponds to a detrital input event (dropstone) at EYC that compromises measurement reliability. (c) Hg accumulation rate profiles for Luitel Lake^30^ and Montcortés Lake,^66^ normalized to the peak value (1940/1945). (d) Historical trends in anthropogenic Hg emissions in Europe, based on data from Streets et al.^63^ (red) and the EDGAR emission inventory^64^ (blue). The EDGAR estimates include emissions from the IPCC climate reference regions Northern Europe (NEU), Western and Central Europe (WCE), and the Mediterranean (MED).^68^ Note that “Europe” is not explicitly defined in Streets et al.,^63^ which may introduce a slight bias when comparing the two inventories.

**Figure 3:**
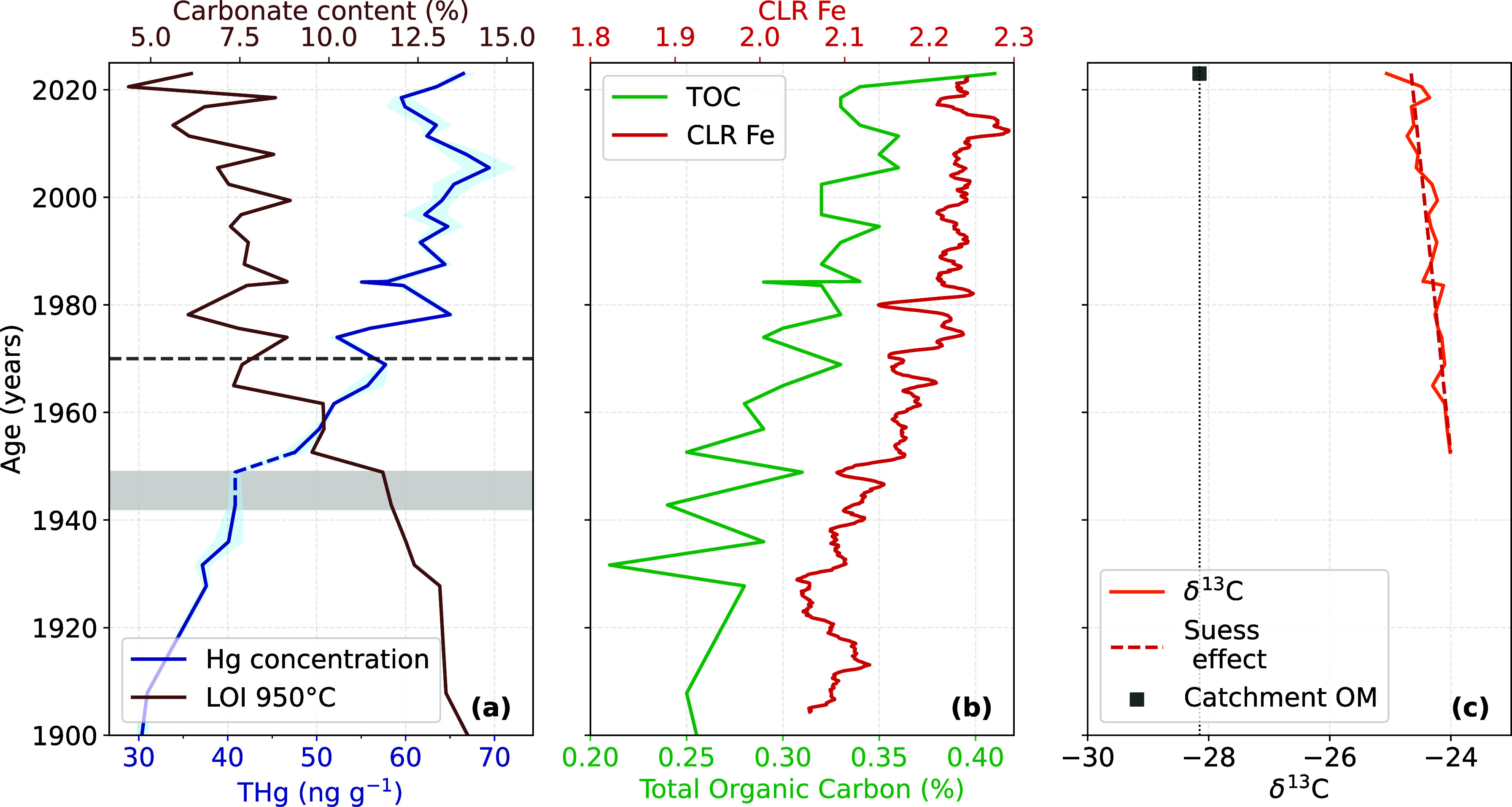
(a) Mercury concentration profile and carbonate content (LOI_950_) for EYC. The shaded area represents measurement uncertainty; the gray shaded area marks the interval perturbed by a dropstone in the sediment. (b) Total organic carbon (TOC, %) and iron centered log-ratio (CLR Fe) profiles for EYC. (c) δ^13^ *C*_org_ values from 1950 to 2023, with a linear trendline highlighting the Suess effect.

**Figure 4:**
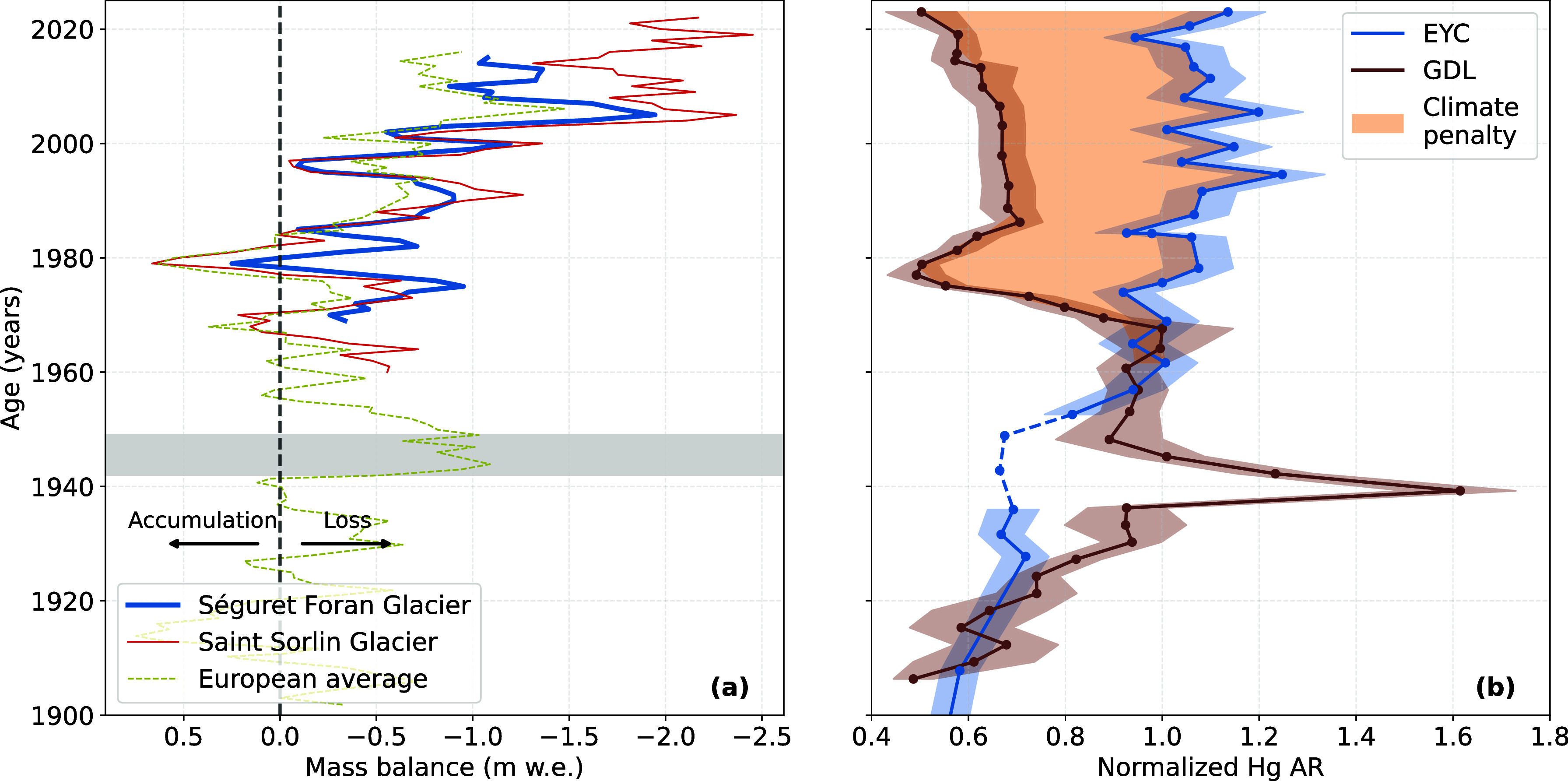
(a) Annual glacier mass balance (millimeter water equivalent) for the Séguret Foran Glacier (deep-learning modeling,^42^ in blue), the Saint-Sorlin Glacier (in red), and the European Alps average (dashed green) based on Huss et al.^21^ The *x*-axis is inverted to highlight the effect of glacier melting on Hg increase. Positive values indicate ice accumulation, while negative values indicate ice loss. The gray area corresponds to the 1942–1949 Great Drought.^83^ (b) Normalized mercury accumulation rate (AR) profiles for the glacier-fed lake (EYC, blue) and the nonglacier-fed lake (GDL, brown), scaled to 1 in 1970 to facilitate comparison. Shaded areas represent the uncertainty associated with the accumulation rates. The dashed line for EYC indicates the section of the core affected by a dropstone. The orange area between the two profiles highlights the climate change-driven penalty.

### Sediment Core Collection and Sampling

Sediment cores were collected in July 2023 using a 90 mm diameter PVC UWITEC pilot corer, deployed from an inflatable zodiac and operated manually with a hammer. This study focuses on core EYC23-01 (IGSN: CNRS0000029549) and GDL23-01 (IGSN: CNRS0000029496), retrieved from Eychauda Lake and Grand Lake, respectively. Sediment cores were retrieved from the deepest locations (22 m for Eychauda Lake and 12 m for Grand Lake), corresponding to the main sediment depocenter, in order to minimize spatial variability and potential sediment focusing effects. For simplicity, these cores are hereafter termed EYC23 and GDL23. All metadata for these two sediment cores are available in the French national core repository https://cybercarotheque.fr/. In the laboratory, each core was split lengthwise into two halves: one designated for chemical analyses and the other reserved for chronology and archived at the EDYTEM repository for long-term preservation (see the Data Availability Statement). Sediment cores were split mechanically lengthwise by a homemade core cutter^43^ and sectioned into 0.5 to 1.0 cm thick slices, depending on facies variations, and then freeze-dried and finely ground using an agate mortar and pestle.

### Dry Bulk Density

The dry bulk density (DBD) was determined by sampling a known volume of wet sediment and then weighing each sample before and after freeze-drying. The loss in mass, corresponding to the water content, was used to calculate the DBD by dividing the weight of the dry sample by the initial sample volume. To reduce variability associated with sampling uncertainties, a 3-point rolling average was applied, yielding a smoother DBD profile and minimizing uncertainties related to the sampling procedure and potential piston effects during coring.

### Chronology

Short-lived radionuclides (^210^Pb, ^226^Ra, ^137^Cs, and ^241^Am activities) were measured by gamma-ray spectrometry on a SAGe well detector located at the Modane Underground Laboratory (LSM, France), a low-background facility.^44^ The EYC23 core was sampled for these gamma measurements. In contrast, the GDL22-01 core had already been dated based on material collected in 2022. Its chronology was established and correlated to that of GDL23-01 using XRF data, as detailed in the Supporting Information (Figure S1). However, the GDL22-01 core lacked sufficient remaining material for comprehensive chemical analyses, prompting collection of the GDL23-01 core. To construct the age–depth models, excess ^210^Pb (i.e., total ^210^Pb minus ^226^Ra) was calculated, and the R package serac^45^ was used. Sediment accumulation rates (SAR) were estimated using the constant flux and constant sedimentation (CFCS) model applied to depth profiles.^46^ Artificial radionuclides ^137^Cs and ^241^Am, which serve as tracers of atmospheric nuclear weapons testing and fallout, provided chronological markers corresponding to the 1963 fallout maximum in the Northern Hemisphere and the 1986 Chernobyl nuclear plant accident.^47^ These markers offer independent validation of the chronology derived from ^210^Pb_ex_.

### Geochemical Analyses

#### Organic Matter and Carbonate Content Analysis

Sediment organic matter (OM, %) and carbonate (CaCO3, %) content were analyzed by loss on ignition (LOI) at 550 and 950 °C, respectively, following the method of Heiri et al.^48^ Briefly, ca. 800 mg of sediment was used to estimate the organic and inorganic carbon contents of the sediments by measuring mass loss at high temperatures in a muffle furnace. Samples were heated at 550 °C for 4 h to estimate organic carbon content (LOI550) and at 950 °C for 2 h to estimate carbonate (inorganic carbon, LOI950) content.

### X-ray Core Scanning

High-resolution X-ray fluorescence (XRF) was carried out on the surface of the split sediment cores on an Avaatech Core Scanner at EDYTEM. Geochemical data were acquired under two tube settings following the protocol of Richter et al.:^49^ (i) 10 kV, 30 s, 1.2 mA for light elements (Al, Si, S, K, Ca, Ti, Mn, and Fe) and (ii) 30 kV, 30 s, 0.75 mA for heavier elements (Cu, Zn, Br, Sr, Rb, Zr, and Pb). Each spectrum was individually deconvoluted to derive relative elemental intensities expressed in counts per second (cps). To address the semiquantitative nature of XRF data, elemental abundances were transformed into Centered Log-Ratios (CLRs).^50^ CLRs were calculated using the following equation

1 $$CLR\left(x\right)=\ln\frac{X_{counts}\left(z\right)}{g\left(z\right)}$$

where *X*_counts_(*z*) is the cps of element *x* at depth *z* and *g*(*z*) is the geometric mean of the selected elements at that depth. The geometric mean was calculated from 12 elements: Al, Si, K, Ca, Ti, Mn, Fe, Zn, Pb, Rb, Sr, and Zr. Log transformation was applied to stabilize variance in the data set, which may partly reflect matrix-related effects such as grain size, water content, or organic matter content variability.^51^

### FTIR-ATR Analysis

Sediment samples were analyzed by Fourier transform infrared spectroscopy with attenuated total reflectance (FTIR-ATR) using a Thermo Scientific Nicolet iS50. Spectra were acquired in the mid-infrared range (4000–400 cm^–1^) at 0.5 cm^–1^ resolution, averaging 60 scans per sample. The instrument was cleaned between runs, and a new background spectrum was recorded for every five samples.

To minimize noise and artifacts (e.g., scattering, reflection, instrumental drift), baseline correction (rubber banding) and Standard Normal Variate (SNV) normalization were applied by using Orange software. Following the published protocol,^52^ a transposed Principal Component Analysis (tPCA) was conducted on the full MIR spectra, treating wavenumbers as variables and samples as observations. This unbiased approach avoids preselecting specific bands or absorbance values.

The tPCA was performed without varimax rotation. In this framework, scores are associated with wavenumbers so a scores’ spectrum can be produced for each principal component, showing peaks that can be attributed to specific compounds present in the sediment sample. Meanwhile, loadings reflect how much of the MIR spectral signal of each sample is accounted for by that each principal component (i.e., the contribution of a given compound to the MIR spectrum in each sample).^52^

### δ^13^ *C*_org_ Analysis

The δ^13^ *C*_org_ of bulk organic matter in sediments was measured using Cavity Ring-Down Spectrometry (CRDS, Picarro Inc.) coupled with a Combustion Module (Costech Inc.) (CM-CRDS) at the geochemistry–mineralogy platform of ISTerre. Prior to analysis, sediment samples were decarbonated by mild acid treatment, dried, and packed into silver and tin capsules. Results were calibrated against the USGS-MAG-1 standard. Analytical methods, calibration, and sample preparation (including decarbonation) are detailed elsewhere.^53,54^

### Total Hg Concentration

THg concentrations were measured using a Direct Mercury Analyzer (DMA-80, Milestone, Italy), following the protocol described by Melendez-Perez et al.^55^ Accuracy was assessed using repeated analyses of the certified reference material ERM-CC141 (soil) (*n* = 37). Measured concentrations (82 ± 5 ng g^–1^) were always within the certified range (83 ± 17 ng g^–1^), yielding a relative standard deviation (RSD) of 6.7% and an accuracy of 99%.

All samples were analyzed in triplicate to assess the measurement precision. The Hg data set reported in this manuscript is presented as mean ± SD obtained from replicate sample analysis and the number of observations (*n*). Additional analytical details are provided in the Supporting Information (Hg analysis section).

### Hg Accumulation Rate

The Hg accumulation rate, Hg AR in μg/m^2^/y, was calculated by using the following equation. It reflects the amount of Hg deposited on the sediment surface over a given area and time period.

2 HgAR=THg·SAR·DBD

In this equation, THg represents the total Hg concentration (μg/g), SAR is the sediment accumulation rate (m/y) from the age model, and DBD is the dry bulk density of the sediment (g/m^3^). The uncertainty in the accumulation rate was determined by propagating the relative errors of each component according to the following equation

3 $$e_{AR}=\sqrt{e_{Hg}^{2}+e_{SAR}^{2}+e_{DBD}^{2}}$$

This relative error was then multiplied by the calculated accumulation rate to determine the absolute uncertainty.

### Comparison of High-Resolution and Low-Resolution Data

A comparison between high-resolution and low-resolution data sets was necessary due to the difference in sampling resolution: X-ray core scanning was performed at 0.5 mm intervals, while the Hg measurements were done at 0.5 cm intervals for GDL and approximately 1 cm for EYC. To align these data sets, the CLR values from the X-ray scans were averaged over each corresponding 0.5–1.5 cm sampling interval. This approach enabled the calculation of linear regressions between the Hg accumulation rates and geochemical proxies.

## Results and Discussion

### Sedimentology and Chronology

The CFCS model applied to the ^210^Pb_ex_ activity profile of core GDL22 defines a well-constrained linear age–depth relationship, with a mean sediment accumulation rate (SAR) of 1.68 mm yr^–1^. This relatively low rate indicates limited input from catchment erosion, as also illustrated in Figure S3.

In contrast, core EYC23 shows a more disturbed ^210^Pb_ex_ profile, likely influenced by glacial activity and instantaneous event deposits.^45^ When these deposits are removed, the CFCS model estimates a SAR of 3.03 mm yr^–1^, reflecting higher input from catchment erosion (Figure S4). Given the complexity of the sedimentation processes in EYC23 (marked by temporal variability linked to glacial and hydrological dynamics), we determined that the CRS age model would be more appropriate than the CFCS approach, which assumes a constant SAR. This choice allows us to better account for fluctuations in sedimentation rates over time. The two normally graded event deposits are dated to 1970 and 1980. All these disturbances are likely linked to flash floods.^38^

The presence of ^137^Cs and ^241^Am peaks further confirms the chronology and provides independent validation for both cores (Figures S3 and S4).

### Anthropogenic Hg Deposition Dominates the Signal Recorded in the Nonglacier-Fed Lake

Depth profiles of total mercury (THg) for both lakes (Figure 2a,b) follow trends similar to accumulation rates (Hg AR), indicating that variations in dry bulk density (DBD) and sedimentation rates have little effect on the Hg AR. THg concentrations are higher in GDL (191.2 ± 47.2 ng g^–1^) than in EYC (53.6 ± 12.3 ng g^–1^), reflecting the higher organic matter (OM) content in GDL sediments (10–12% LOI550) compared with the more minerogenic, OM-poor EYC sediments (2–3%). When Hg concentrations are normalized to LOI550, the two profiles become comparable (Figure S5), showing that the observed difference in THg largely arises from the roughly 4-fold difference in OM content between the catchments.

To assess the contribution of lake catchment erosion input to the lake Hg record, OM content was used as a proxy of soil OM leaching, and potassium (K), titanium (Ti), and zirconium (Zr) were used as detrital proxies because they originate from physical rock erosion and are geochemically stable in sediments.^38,56,57^ In the case of GDL, no significant correlations were found between Hg AR and detrital proxies (Figure S6), nor with OM content (*r* = −0.212 *p* = 0.67, Figure S7). This result suggests that particulate inputs from soil erosion and variations in primary productivity had a limited influence on the observed Hg profile. This also indicates that the high organic matter content in GDL has not limited the scavenging of atmospheric Hg, whether of anthropogenic or natural origin, over the past century.^58^

The Hg accumulation profile at GDL shows a notable peak in the 1940s, likely linked to increased industrial activity during World War II.^30^ A broader and slightly less pronounced peak extends from the 1950s to the 1970s, coinciding with the regional expansion of coal combustion and cement production.^30^ This is followed by a marked decline in Hg accumulation rates beginning in the 1970s, attributed to the reduction in Hg emissions due to stricter industrial regulations and the co-benefits of sulfur control policies.^59,60^ Hg levels subsequently dropped to values comparable to those at the start of the century. Notably, a more recent decrease from 2010 to 2023 may reflect global efforts driven by the Minamata Convention to phase out Hg use and reduce emissions.^61,62^ However, the low sedimentation rate at the GDL results in limited temporal resolution for this period, with only two data points available, precluding any assessment of recent emission changes.

The GDL sediment record closely aligns with the historical pattern of anthropogenic Hg emissions in Europe (Figure 2d). It exhibits a clear mid-20th-century peak, followed by a sharp decline, closely matching the emissions trajectory reported by Streets et al.,^63^ which shows European emissions peaking in the 1970s. However, the EDGAR inventory^64^ presents a somewhat different picture, with a broader and less intense peak and a more gradual decline (Figure 2d). There is ongoing debate in the scientific community regarding the timing and magnitude of peak Hg emissions.^65^ The clear midcentury peak observed in the GDL sediment record supports the trend indicated by the Streets inventory and highlights the value of well-dated sediment records as independent archives of past atmospheric emissions.

These observations are consistent with findings from other studies in Europe. For example, Guédron et al.^30^ reported similar patterns at Luitel Lake (Figure 2c), located roughly 60 km from GDL (see Figure 1) and more directly impacted by emissions from the Grenoble metropolitan area (714,799 inhabitants). Comparable results were also reported at Montcortés lake in the Pyrenees^66^ (Figure 2c), where Hg accumulation rates increased significantly after 1840—marking the onset of the Industrial Era—and rose 5-fold during World War II, largely due to intensive coal use, the global rise in Hg production, and mining activity at Almadén, Spain.^66^ In the following decades, a steady decline in Hg accumulation rates was observed, which is consistent with broader Western Mediterranean trends. This decrease is consistent with the estimates by Zang et al.,^62^ who reported a 1–2% annual reduction in emissions since the 1990s—a trend also observed in records from Luitel Lake, Montcortés Lake, and multiple peat cores in northwestern Spain.^67^

It is worth mentioning that GDL records a combination of a regional Hg signal -linked to pollution from the Grenoble valley, as clearly described by the sediment core from Lake Luitel^30^ and broader European trends. This finding supports the conclusion that the Hg accumulation rate recorded in the GDL sediment core predominantly reflects atmospheric deposition. As such, it offers a reliable archive for evaluating long-term trends in atmospheric Hg inputs and for comparison with historical emission inventories.

In the EYC Hg record (Figure 2b), Hg AR exhibits a broadly similar trend to that for GDL prior to the 1970s, with the notable exception of a distinct peak during World War II present in the GDL record but absent in EYC. Between 24.3 and 28 cm, a large rock disrupted the sedimentary sequence, preventing higher-resolution sampling (see also Figure S4). This rock is likely a dropstone deposited during a snow avalanche onto the frozen lake, later transported across the surface by drifting ice and released to the lake floor during melting^69^ (Event A, gray band in Figure 2b). This event disturbed the sediment structure and likely obscured the short-lived signal from the 1940s. Then, from the 1950s through the 1970s, both sites converge and display a general increase in Hg ARs, consistent with reported anthropogenic Hg emissions across Europe during this period (Figure 2d).^63,64^ From the 1970s onward, the two records diverge; Hg ARs decrease in the GDL record, in line with emission inventories, while they exhibit a renewed increase for EYC. This divergence points to the influence of an additional source or process affecting Hg ARs at EYC. Since the primary difference between the two lakes is the presence of a glacier at EYC, it is reasonable to hypothesize that glacier melt may contribute to the post-1970s Hg signal. However, a comprehensive assessment of all potential sources, summarized in Figure 1, is necessary to support this interpretation.

### Influence of Erosion and Organic Matter on Mercury Accumulation in the Glacier-Fed Lake

To evaluate erosion-driven Hg inputs from the watershed, several mineral samples were analyzed to determine background Hg concentrations (Tab. S4). These samples show an average of 29.0 ± 2.7 ng g^–1^, fully consistent with concentrations at the bottom of the sediment core (pre-1920; WWI). This indicates that the biogeochemical background of the Eychauda watershed is about 30 ng g^–1^ and that the elevated Hg concentrations at the top of the core (65 ng g^–1^; Figure 2b) cannot be explained by enhanced erosion following glacial retreat as the catchment is mostly constituted by marls and limestone, most probably in a relatively coarse fraction (Figure S2).

Carbonate content in the EYC core decreases as glacial input declines, reflecting reduced bedrock abrasion by the glacier and a lower transport of silty to sandy material to the lake (see Figure 3a).^70^ This trend coincides with the rising atmospheric Hg concentrations and deposition. Between 1920 and 1970, Hg concentrations and carbonate content show a strong anticorrelation (*r* = – 0.984, *p* = 7.73 × 10^–12^; Figure 3a). At first glance, this might suggest a dilution effect, where a lower carbonate input leads to apparently higher Hg concentrations. However, sedimentation rates increased only slightly after 1970 (Figure S4), meaning that simple dilution would produce the opposite effect, i.e., lower Hg concentrations where sedimentation is higher.

Thus, two processes likely act simultaneously: (i) a decline in carbonate content due to reduced bedrock erosion and (ii) an increase in fine-grained particulate input associated with glacier melting, which is relatively enriched in Hg. Therefore, the decrease in carbonates reflects reduced glacial flour input, while the concurrent increase in Hg corresponds to enhanced glacier-derived Hg mobilization and ongoing atmospheric deposition.

The OM record provides additional insight into the pathways through which Hg reaches and accumulates in the lake sediment. Total organic carbon (TOC) remains low but gradually increases from 0.21% in 1910 to 0.41% in 2023, consistent with the low productivity and sparse vegetation of the watershed (Figures S2 and S11). Hg concentrations in watershed vegetation averaged 28.5 ± 22.9 ng g^–1^ and in the lake biofilm averaged 32.8 ng g^–1^, both near geochemical background levels. The highest value, 54.5 ng g^–1^, was measured in moss, which accumulated more Hg than the other sampled species.^71^

Lake sediment δ^13^ *C*_org_ (−24.4 ± 0.2‰) Figure 3c) values remained stable from 1950 to 2023 and were significantly higher than lake watershed OM δ^13^ *C*_org_(−28.2 ± 0.4‰, *n* = 4, see Tab. S4), indicating a primarily in-lake OM production that did not change substantially over time. The slight decrease in δ^13^ *C*_org_, with a slope of −0.012‰ yr^–1^, is consistent with the Suess effect (the δ^13^C dilution of atmospheric carbon dioxide from fossil fuel burning) and agrees with literature data.^72^ In parallel, Hg concentrations in watershed vegetation averaged 28.5 ± 22.9 ng g^–^1 (*n* = 4), near geochemical background levels. The highest value, 54.5 ng g^–1^, was measured in moss, which is known to accumulate more Hg than the other sampled species.^71^ These observations rule out a significant contribution from the export of Hg bound to terrestrial OM and indicate that although the primary productivity of the lake appears to be increasing slightly over time, the increase in Hg after 1970 cannot be entirely explained by the lake’s biological pump.

TOC and Fe are strongly correlated (*r* = 0.912, *p* < 0.01), as are TOC and Hg (*r* = 0.757, *p* < 0.01). Clays also show a strong correlation with TOC (*r* = 0.880, *p* < 0.01) (Figure S13). These Fe- and clay-rich particles likely correspond to the finest particles available in recently deglaciated areas and transported to the lake.^73,74^ The comobilization of Fe and Hg suggests shared geochemical controls and indicates that glacial retreat enhanced the flux of Hg-rich Fe and clay particles into the lake. This interpretation is consistent with previous studies reporting elevated Hg^17,19,75,76^ and Fe^77–79^ concentrations in glacial meltwaters. Overall, the strong correlations between Hg, OM, Fe, and clay minerals support the hypothesis that fine mineral particles act as the primary carriers of glacier-derived Hg into the lake, in combination with autochthonous OM, which acts as a biological pump and scavenger and promotes the transfer of Hg to the lake sediment^58,80–82^

### Impact of Glacier Retreat

In order to assess the glacier’s impact on Hg accumulation in lake sediments, we investigated the behavior of the Séguret Foran Glacier (SEG). In Figure 4a, surface mass-balance data for SEG, derived from a deep-learning approach,^42^ are compared with direct in situ observations from the nearby Saint-Sorlin Glacier (SOR), located within 40 km and at comparable elevations (2900–3300 m; see Figure 1). SOR serves as a reasonable analog, generally following the Alpine average across the European Alps^21^ but exhibiting a more pronounced mass loss due to its smaller size and lower altitude. The annual surface mass balance evolution for glaciers in the European Alps shows strongly negative values during 1942–1949, followed by a rebound between 1955 and 1970, and a subsequent general decrease to the present. These results indicate that SEG’s behavior is consistent with regional and European patterns. Interestingly, Hg accmulation rates correlate well with SEG mass balance (*r* = −0.658, *p* < 0.001 see Figure 4b) when a 1 year lag is applied to account for the time required for glacial signals to appear in the sediments. This anticorrelation indicates that peaks in Hg AR tend to occur during years of strongly negative mass balance, highlighting the significant influence of glacier dynamics on sedimentary Hg inputs.

### Glacier Hg Mass Balance

The evidence presented above strongly indicates that glacier-derived Hg is the sole source capable of explaining the post-1970 increase in Hg recorded in the EYC sediments. To test this hypothesis quantitatively, we applied a simple mass-balance approach to estimate the Hg contribution from a glacier melt.

Figure 4b shows the Hg AR profiles for EYC and GDL normalized to their respective 1970 peak values. From 1970 onward, the two profiles diverge: the brown curve (GDL) reflects regional atmospheric deposition, while the EYC curve captures both atmospheric inputs and glacier-derived contributions. Subtracting the GDL profile from the EYC profile isolates the glacier melt signal, interpreted here as the climate change-driven penalty. Integrating the area between the two curves and multiplying by the glacier surface area and by the 1970 peak Hg AR provides an estimate of the total Hg mass released from glacier melt and accumulated in the sediments between 1970 and 2023. Propagating uncertainties using a Monte Carlo approach yields a total mass of 415 ± 146 g (details in the Supporting Information).

To further constrain this estimate, the volume of ice lost from 1970 to present was calculated using satellite-derived glacier surface areas,^84^ ^,^ ^87^ combined with mass-balance data.^42^ The total ice loss corresponds to approximately 36 million m^3^ of water equivalent. Assuming dissolved Hg concentrations of 1–5 ng L^–1^, typical concentrations of ice and snow,^86^ this translates to 36–180 g of dissolved Hg, representing 8–43% of the estimated total Hg released.^85^

Particulate Hg stored in cryoconite deposits was also taken into account. Cryoconite consists of dark aggregates of mineral particles and organic matter that accumulate on glacier surfaces. By reducing the surface albedo, these deposits locally enhance melting and promote the concentration of particles and impurities. Cryoconite holes also host active microbial communities (e.g., bacteria and algae), which can further facilitate the accumulation and retention of metals and other contaminants.^87,88^ Previous studies have reported Hg concentrations of 150–300 ng g^–1^ in cryoconite.^87,89^ To estimate the Hg content associated with cryoconite at EYC, we applied the approach of Huang et al.^29^

4 $$P\left(t\right)=C_{Hg}WfS\left(t\right)$$

where *P*(*t*) is the annual amount of Hg stored in cryoconite (grams per year of formation), *C*_Hg_ is the Hg concentration in cryoconite (ng g^–1^), *W* is the cryoconite mass per m^2^, *f* is the fraction of glacier area covered by cryoconite, and *S*(*t*) is the glacier surface area at time *t*. The total Hg stored over the last 53 years is obtained by integration

5 $$P_{tot}=\int_{1970}^{2023}C_{Hg}WfS\left(t\right)dt=C_{Hg}Wf\int_{1970}^{2023}S\left(t\right)dt$$

Assuming constant *C*_Hg_, *W*, and *f* over time and using glacier surface-area data, we estimate that 120–240 g of Hg could have accumulated in cryoconite between 1970 and 2023 (details in the Supporting Information). When combined with the dissolved Hg estimates (36–180 g), this yields a total Hg release of 288 ± 132 g, consistent with the 415 ± 146 g inferred from the sediment record; this means that secondary mercury from glacier melting could explain 70% of the Hg we expect.

This calculation necessarily involves numerous assumptions and extrapolations. For example, *W* values are derived from Himalayan glaciers and may underestimate cryoconite accumulation in the Alps; likewise, the adopted 6% cryoconite coverage fraction (*f*) comes from Arctic studies and may be higher for alpine glaciers. Nonetheless, this first-order estimate strongly supports the hypothesis that the increase in Hg recorded in EYC sediments is linked to glacial melt and reflects a regional climate change-driven penalty. While contributions from watershed erosion cannot be entirely excluded, the temporal alignment between Hg accumulation, glacier mass balance, and the glacier’s estimated residence time (100 years) indicates that a substantial fraction of the excess Hg originates from historically deposited Hg released during glacier melt. Since 1970, the Hg accumulation rate in Eychauda Lake has been approximately 50% higher than that in the reference lake, illustrating the magnitude of this effect.

Direct meltwater and cryoconite sampling at the Seguret Foran Glacier was not possible—by July 2023, the glacier had almost completely disappeared (see Figure S11). As a result, our estimates are based on literature values and regional glacier data sets. While the estimates based on sediment records and ice loss calculations show good agreement, we acknowledge the uncertainty introduced by extrapolating parameters from other glacial systems and the absence of direct meltwater sampling from the Séguret Foran Glacier. However, the Séguret Foran Glacier has been shown to behave comparably to other European alpine glaciers, lending confidence to our assumptions. The sediment record therefore provides compelling evidence that glacial melt has played a major role in recent Hg dynamics at this site and explains the divergence observed between the two lakes after 1970, despite the decline in regional atmospheric emissions recorded in emission inventories and in the nonglacier-fed Grand Lake sediment archive.

### Broader Implications and Policy Relevance

Although this study focuses on a specific glacial system in the Alps, the mechanism described is relevant across the European Alps. Current estimates indicate that the Alps may lose roughly 100 km^3^ of ice by the end of this century.^21^ On the assumption of a mean Hg content in glacier ice, dissolved, particulate, and cryoconite fractions, on the order of 10 ng L^–1^, this would correspond more or less to 1000 kg of Hg being potentially released by Alpine glacier melting alone. This estimate is consistent with figures reported in previous studies.^90^ Although small in area, the implications for long-term global Hg budgets and the efficacy of policy are significant and underscore the need for including climate-driven remobilization of Hg within global models and mitigation strategies.^13,65,91^

While this study focuses on environmental trends in total Hg driven by cryospheric change, future work should investigate mercury speciation in glacier-fed lake sediments. Recent studies from proglacial systems on the Tibetan Plateau suggest that changes in dissolved organic matter (DOM) composition can stimulate microbial activity and potentially favor the development of Hg-methylating communities.^92^ Such processes have been linked to increased methylmercury (MeHg) production relative to total Hg in proglacial lake waters. By analogy, glacially remobilized Hg in our study region may also be susceptible to methylation under suitable biogeochemical conditions, with important implications for Hg bioavailability and ecological risk. Integrating Hg speciation, DOM dynamics, and microbial processes therefore represents a key direction for future research in glacial-fed aquatic ecosystems under ongoing climate change.

Glaciers accumulate and offer lagged sources of contaminants. Systems like Grand Lake respond rapidly to anthropogenic forcing, whereas glacier-fed systems like Eychauda Lake are more inert and reflect the imprint of climate change and pollution with a time delay. This buffer response diminishes the instantaneous pressure of emissions but postpones ecosystem recovery. These dynamics are the core of policy success: effects of work under the Minamata Convention will span decades or generations to actualize in terms of measurable reductions in Hg levels in glacier-fed ecosystems. Accounting for this lag factor in policy assessment is essential in order to set realistic timelines and not misplace optimism for near-term policy success.

Reduction of greenhouse gases slows down global warming and glacier melting but, as a cobenefit, also limits release of contaminants such as Hg.^93^ The approach employed in this study can be applied to other vulnerable environments, such as thawing permafrost^94^ and vulnerable Arctic or coastal systems where disturbed biogeochemical conditions can remobilize mercury as well. Sediment records from such climate-sensitive settings are a powerful tool for assessing the efficacy of emissions reduction and the early stage influence of climate change on Hg cycling.^7,95^ These records highlight the intrinsic link between climate policy and pollutant control.

## Supplementary Material



## Data Availability

The dating and chemical analysis results of the sediment cores and the database, along with the Python scripts used to generate the figures in this study, are available in Zenodo 10.5281/zenodo.17549863. All sediment cores collected as part of this project are cataloged in the Cyber Carothèque Nationale https://cybercarotheque.fr under the mission name EPOCH-ALPS23. The archived half-cores are preserved to ensure long-term accessibility and are available for future verification, complementary analyses, or use by other researchers, thereby supporting the integrity and transparency of the data set. Data for the Saint Sorlin and Sarenne glaciers are available at the following link: https://glacioclim.osug.fr/Donnees-des-Alpes.

## Notes

Core dating and age–depth translation; Hg analytical procedures and quality control (LOD, LOQ, RSD, accuracy); estimation of excess sedimentary Hg linked to the climate change-driven penalty; Monte Carlo uncertainty analysis; glacier mass-balance calculations; dissolved Hg release estimates; cryoconite Hg mass-balance modeling; uncertainty assessment; supplementary tables (S1–S7); supplementary figures (S1–S13); and supplementary references (PDF)
