# Supplementary material for: Glacier Melt as a Source of Mercury: Implications for Ecosystem Recovery and Environmental Trends
Source: Environ Sci Technol. 2026 Apr 15;60(16):12182–93. doi: 10.1021/acs.est.5c16308 (PMC13181719; doi:10.1021/acs.est.5c16308)
Supplement: Supplementary file 1 [file es5c16308_si_001.pdf]

Supporting information (SI) for:

Glacier melt as a source of mercury: Implications for ecosystem recovery and environmental trends.

Davide Mattio,*,† Stéphane Guédron,‡ Pierre Sabatier,¶ Yann Bertrand,† Nicolas Bonfanti,¶,§ Sylvain Campillo,‡ Aurélien Dommergue,† David Gateuille,¶ Elsa Gautier,† Antonio Martínez Cortizas,‖ Emmanuel Naffrechoux,¶ Antoine Rabatel,† and Hélène Angot*,†

†*Univ. Grenoble Alpes, CNRS, INRAE, IRD, Grenoble INP, IGE, Grenoble, 38400, France*

‡*ISTerre, Univ. Grenoble Alpes, Université Savoie Mont Blanc, CNRS, IRD, UGE, Grenoble, 38610, France*

¶*EDYTEM, CNRS, Université Savoie Mont Blanc, Le Bourget du Lac, 73376, France*

§*Université Savoie Mont Blanc, INRAE, CARRTEL, Thonon-Les-Bains, 74200, France*

‖*EcoPast (GI-1553), Facultade de Bioloxía, Universidade de Santiago de Compostela, 15782 Santiago de Compostela, Spain*

E-mail: davide.mattio@univ-grenoble-alpes.fr; helene.angot@univ-grenoble-alpes.fr

Contents

- S1 - Core dating
- S2 - Hg analysis
- S3 - Estimation of sedimentary mercury linked to the climate change-driven penalty
- S4 - Estimation of mercury released from glacial melt

S1 - Core dating

Additional information related to dating: EYC23 was directly dated at the LSM, while GDL23 was not dated. Instead, the dating of GDL22 (collected during a previous field campaign for a different project) was used, and a comparison was made by utilizing X-ray data to align the two sediment cores and infer the age of the layers in GDL23 (see Fig. S1).

Translation of the dating

The dating methodology for the 2023 core relies on the following premise: points with identical elemental compositions are considered equivalent and thus presumed to share the same age. Once some common points on the two cores have been selected, the next step is to calculate the *equivalent sedimentation rate* (Γ), which is crucial for dating the core. The equation for Γ is as follows:

$$\Gamma = \frac{\Delta h}{t_0 - t_1} \tag{S1}$$

where Δh represents the difference between two points in the 2023 core, and $t_0 - t_1$ denotes the difference between the ages of the points examined, referring to the datation of the 2022 core. In Table S1, an example of GDL is provided to demonstrate how this can be calculated.

Once Γ is determined, it should be applied within the associated depth range to obtain the correct age using the following formula:

$$t_1 = t_0 - \frac{\Delta h}{\Gamma} \tag{S2}$$

thus, starting from the age of the upper layer (t_0), one can obtain the age of the underlying layer (t_1) using the thickness of the layer (Δh) and Γ at the corresponding depth. This approach enabled the determination of the age of the various samples.

S2 - Hg analysis

Table S2 presents the measured blank values during the analysis, along with the mean and standard deviation values. These parameters were used to assess the Limit of Detection (LOD) and Limit of Quantification (LOQ) of the measurement method. The LOD was determined using:

$$\text{LOD} = \bar{x}_b + 3s_b \tag{S3}$$

while the LOQ was evaluated using:

$$\text{LOQ} = \bar{x}_b + 10s_b \tag{S4}$$

where $\bar{x}_b$ and s_b stand for mean value of blanks and standard deviation of the blanks, respectively.

In the Table S3 we can find the results of the various measurements on the sample certified ERM® CC-141. With this data it was possible to calculate the relative standard deviation (RSD):

$$\text{RSD} = \frac{\bar{x}}{s} \tag{S5}$$

and the accuracy, considering that the sample is certified with a concentration of 83 ± 17 ng/g of Hg.

$$\text{Accuracy} = \frac{\bar{x}}{83} \tag{S6}$$

where $\bar{x}$ and s stand for mean of ERM concentration measured and standard deviation of ERM, respectively.

S3 - Estimation of sedimentary mercury linked to the climate change-driven penalty

The excess mercury (Hg) flux attributable to glacial melt was calculated by comparing the sedimentary Hg accumulation rate (HgAR) at the glacier-fed lake (EYC) with a nearby non-glacier-fed reference lake (Grand Lake, GDL) reflecting changes in regional atmospheric deposition. Both HgAR profiles were normalized to their respective values in 1970. The integrated area between the normalized curves over the 1970–2023 period provides the cumulative excess flux due to glacial input:

$$\mathrm{A}_{\text{EYC-GDL}} = \int_{1970}^{2023} \Big(\mathrm{HgAR}_{\mathrm{EYC}}^{\mathrm{norm}}(t) - \mathrm{HgAR}_{\mathrm{GDL}}^{\mathrm{norm}}(t) \Big)\, dt \tag{S7}$$

The calculated normalized integrals were:

$$\int_{1970}^{2023} \mathrm{HgAR}_{\mathrm{EYC}}^{\mathrm{norm}}(t)\, dt = 53.60\ \mathrm{yr}, \quad \int_{1970}^{2023} \mathrm{HgAR}_{\mathrm{GDL}}^{\mathrm{norm}}(t)\, dt = 39.22\ \mathrm{yr} \tag{S8}$$

yielding a net area of 14.38 yr

The excess Hg mass was then obtained by multiplying the net area by the reference HgAR in 1970 and by the effective sedimentation area of the EYC lake. This effective area was derived from the lake's bathymetry by excluding the steep slopes where sediment accumulation is negligible, thereby restricting the calculation to the depositional zone, estimated to 10 ha.

$$\begin{aligned} M_{\mathrm{Hg,glacier}} =& \mathrm{A}_{\text{EYC-GDL}} \cdot \mathrm{HgAR}_{\mathrm{EYC}}(1970) \cdot S_{\mathrm{EYC}} = \\ & 14.38\,\mathrm{y} \cdot 290\,\mu\mathrm{g/m^2/y} \cdot 100000\,\mathrm{m}^2 = 417\,\mathrm{g} \end{aligned} \tag{S9}$$

This amount represents the mercury attributable to the climate change-driven penalty, i.e., the amount of mercury released due to cryospheric changes in the Eychauda catchment

and accumulated in lake sediments between 1970 and 2023 (orange area in Fig. 4(b)).

To account for uncertainties in HgAR values in both lakes, a Monte Carlo approach was applied. Randomly sampling HgAR values within their respective absolute errors (assuming Gaussian distributions), the normalized integrals and net area were recalculated for 10,000 iterations. This yielded an excess Hg mass of:

$$M_{\mathrm{Hg,glacier}} = 415 \pm 146 \text{ g} \tag{S10}$$

The Monte Carlo result confirms the deterministic estimate (417 g) while providing a quantified uncertainty due to measurement errors in HgAR. The relatively large standard deviation reflects the propagation of errors from both lakes' HgAR, highlighting the variability in the estimate of glacier-released Hg.

S4 - Estimation of mercury released from glacial melt

To estimate the total mass of mercury (Hg) potentially released from melting glacier ice, we used a mass-balance approach combining glacier surface mass-balance data, multitemporal inventories of glacier surface area, and literature values of Hg concentrations in glacial ice.

Ice volume loss (1970–2022)

The cumulative ice loss over the period 1970-2022 was calculated from the annual surface mass balance of the Séguret Foran Glacier[1] multiplied by its surface area. Glacier surface areas from 1970 to 2022 were derived from satellite observations topographic maps and aerial photographs;[2,3] missing years were linearly interpolated. This procedure yielded an estimated total ice loss between 1970 and 2022 of:

$$\Delta V_{\text{ice}} = 3.6 \times 10^7 \text{ m}^3 \quad \text{(water equivalent)} \tag{S11}$$

Mercury concentration in glacier ice

Based on values reported for remote glacial environments, we assumed a dissolved Hg concentration of 1–5 ng L^{-1} in glacier ice.[4,5]

$$M_{\text{Hg,dissolved}} = \Delta V_{\text{ice}} \cdot C_{\text{Hg}} \tag{S12}$$

Using $\Delta V_{\text{ice}} = 3.6 \times 10^7 \text{ m}^3 = 3.6 \times 10^{10}$ L at 1 ng L^{-1}:

$$M_{\text{Hg,dissolved}} = 3.6 \times 10^{10} \cdot 1 = 3.6 \times 10^{10} \text{ ng} = \mathbf{36\ g} \tag{S13}$$

At 5 ng L^{-1}:

$$M_{\text{Hg,dissolved}} = 3.6 \times 10^{10} \cdot 5 = 1.8 \times 10^{11} \text{ ng} = \mathbf{180\ g} \tag{S14}$$

Estimation of Hg stored in cryoconite

To estimate the total mass of Hg stored in cryoconite on the Séguret Foran Glacier between 1970 and 2023 we used the approach of Huang et al.[6]

The annual Hg mass in cryoconite at time t was calculated as:

$$P(t) = C_{\mathrm{Hg}}\, W\, f\, S(t), \tag{S15}$$

where:

- C_{Hg} = Hg concentration in cryoconite (ng g^{-1}),
- W = mass of cryoconite per unit area (g m^{-2}),
- f = fraction of the glacier surface covered by cryoconite,
- $S(t)$ = glacier surface area at time t (m^2).

The cumulative Hg stored over the study period is then:

$$P_{\mathrm{tot}} = \int_{1970}^{2023} C_{\mathrm{Hg}}\, W\, f\, S(t)\, dt = C_{\mathrm{Hg}}\, W\, f \int_{1970}^{2023} S(t)\, dt. \tag{S16}$$

Parameter values and assumptions

- **Hg concentration in cryoconite (C_{Hg}):** We adopted a conservative range of 150–300 ng g^{-1} based on alpine studies.[7]
- **Mass of cryoconite per m^2 (W):** Taken as 300 g m^{-2}, the mid-range of values reported for Himalayan glaciers (292–334 g m^{-2}).[8,9]
- **Fraction of surface covered by cryoconite (f):** We used 6%, corresponding to the maximum value observed on polar glaciers, which likely provides a lower bound for alpine glaciers.

- **Glacier surface area ($S(t)$):** Derived from multitemporal inventories of glacier surface-area of Séguret Foran Glacier from 1970 to 2022, with linear interpolation for missing years. The cumulative area-integrated time term $\int_{1970}^{2023} S(t)dt$ corresponds to approximately 45×10^7 m^2·years

Results

Applying the above parameters yields:

120–240 g Hg stored in cryoconite between 1970 and 2023.

Adding the dissolved-Hg estimate (36–180 g) yields a total of 156–420 g, consistent with the observed excess Hg in EYC sediments over the same period (416 ± 146 g).

Uncertainties

Several sources of uncertainty affect this estimate:

- W values are derived from Himalayan rather than alpine glaciers.
- The adopted f value comes from Arctic glaciers; coverage may be higher on alpine glaciers.
- C_{Hg} is assumed constant over time and across the glacier, which may underestimate temporal or spatial variability.

Despite these limitations, the calculation provides a first-order estimate consistent with the sedimentary record, supporting the hypothesis that glacial melt and cryoconite release have driven the post-1970 increase in Hg at Lake Eychauda.

All datasets used in these calculations, including glacier surface mass-balance data and the glacier surface areas from the multi-temporal inventories are provided in the Data Availability Statement.

Table S1: Calculation of the equivalent sedimentation rate for the GDL sediment core.

h **2022 (cm)**	h **equiv. 2023 (cm)**	**t (y)**	Γ **(cm/y)**
0.0	0.0	2022.00	0.382
1.1	3.9	2011.80	0.108
2.2	5.0	2001.59	0.151
2.7	5.7	1996.95	0.135
3.5	6.7	1989.53	0.180
4.1	7.7	1983.97	0.086
5.1	8.5	1974.69	0.033
5.5	8.7	1968.58	0.026
6.3	9.4	1941.91	0.033
7.2	10.4	1911.92	0.054
7.7	11.3	1895.25	0.030
9.3	12.9	1841.92	

Table S2: Measurements of blank samples taken during several days of analysis. For each day (the columns), the initial analysis and any measurements resulting in a zero quantity of Hg (highlighted in red) were excluded from the calculation of the average ($\bar{x}_b$) and standard deviation (s_b). Note that the first daily measured value is typically excluded as part of standard procedure to ensure instrument cleanliness before starting the measurements.

Hg mass (ng)						
**0.61**	**0.21**	**0.52**	**0.26**	**0.25**	**0.29**	**0.21**
0.15	0.15	0.32	**0**	0.15	0.15	0.16
0.15	0.15	0.53	0.15	0.15	0.15	**0**
0.15	**0**	0.77	0.16	**0**	0.15	0.16
	0.15	0.15			0.19	0.20
	0.15	0.15				
	0.15	0.15				
	0.15					

$\bar{x}_b$ (ng)	s_b (ng)	LOD (ng)	LOQ (ng)
0.20	0.14	**0.62**	**1.59**

Table S3: Results from the certified sample measured during the analysis rounds are presented below, with each column representing a different day. The data includes mean value ($\bar{x}$), standard deviation (s), RSD and accuracy. In red the values that are outside of the range of the certified concentration

**Hg concentration ERM - CC141 (ng/g)**						
74.77	80.15	88.68	88.68	93.08	86.14	80.06
80.56	84.88	77.67	77.67	80.57	84.39	76.44
85.27	76.90	78.84	78.84	77.32	76.80	88.68
88.28	79.03	81.50	81.50	78.49	77.51	89.93
	86.81	81.30	81.30	81.87	81.37	
		78.40	78.40	77.86	**101.49**	

$\bar{x}$ **(ng)**	s **(ng)**	**RSD**	**Accuracy**
82.2	5.5	**6.7%**	**99%**

Table S4: Soil and vegetation samples collected from the Eychauda Lake watershed. Reported values correspond to total mercury (THg) concentrations, with associated standard deviations (SD) when available. Sampling points (1–5) correspond to those shown on the map (Fig.S2).

**Point**	**Sampling zone**	**Material**	**THg (ng g^{-1})**	**SD**	δ^{13}C
1	Moraine (black shales)	Moraine soil	31.84	0.83	
		Grass vegetation	10.88	–	-28.38
2	Grassland	Grass vegetation	13.33	0.51	-27.89
		Subsoil	28.65	1.70	-27.72
3	Delta (estuary)	Grass leaves	20.19	–	-28.63
		Grass stems	4.51	–	
		Mosses	54.48	–	
		Black soil	26.47	–	
4	Lake	Biofilm on stones	32.80	–	-29.32

Table S5: Characteristics of the two lakes studied: Grand Lake (GDL) and Eychauda Lake (EYC)

**Lake**	**Position**	**Elevation (m)**	**Surface** (km^2)	**Watershed/surface**
GDL	45°2′30″N 6°28′24″E	2282	0.047	83
EYC	44°55′55″N 6°28′39″E	2517	0.151	17

Table S6: Downcore radionuclide activities measured in the Grand Lake (GDL22) sediment core. Activities are expressed in mBq g^{-1}.

top (cm)	bottom (cm)	thickness (cm)	210Pb (mBq/g)	Err 210Pb (mBq/g)	226Ra (mBq/g)	Err 226 Ra (mBq/g)	210Pb ex (mBq/g)	err Pb exc (mBq/g)	241Am (mBq/g)	Err 241Am (mBq/g)	137Cs (mBq/g)	err 137 Cs (mBq/g)
0	0,5	0,5	1022,2	26,8	69,9	2,0	952,3	26,9			55,3	2,0
0,5	1	0,5	984,1	23,6	75,3	1,8	908,8	23,7			59,7	1,9
1	1,5	0,5	733,9	8,4	82,8	0,8	651,1	8,4	1,0	0,2	72,3	0,8
1,5	2	0,5										
2	2,5	0,5	595,3	18,4	92,3	2,0	502,9	18,5	1,5	0,5	63,1	1,9
2,5	3	0,5										
3	3,5	0,5	657,4	19,0	85,7	1,9	571,7	19,1	1,0	0,6	80,4	2,1
3,5	4	0,5	538,0	18,3	82,7	2,0	455,3	18,4	1,1	0,6	102,0	2,5
4	4,65	0,65	385,3	15,4	86,5	1,9	298,9	15,5			109,9	2,4
4,65	5,2	0,55										
5,2	5,7	0,5	362,7	13,6	80,4	1,7	282,3	13,7			227,5	3,2
5,7	6,2	0,5	465,4	16,8	82,9	2,0	382,5	16,9			994,2	7,0
6,2	6,7	0,5	383,4	15,2	86,2	1,9	297,2	15,4			237,4	3,5
6,7	7,35	0,65	413,3	16,0	91,8	2,0	321,6	16,1	2,0	0,6	125,2	2,7
7,35	8,1	0,75	376,6	14,5	94,3	1,9	282,3	14,6	2,7	0,6	184,3	3,0
8,1	8,7	0,6										
8,7	9,25	0,55	331,8	15,7	110,5	2,3	221,3	15,9	5,4	0,7	295,9	4,2
9,25	10	0,75	321,7	8,0	85,5	1,1	236,2	8,1	1,4	0,3	98,0	1,3
10	10,5	0,5	279,2	13,0	89,7	1,9	189,5	13,1			41,0	1,5
10,5	11	0,5	248,5	5,7	93,5	0,9	155,0	5,8	0,7	0,2	21,1	0,5
11	11,5	0,5	213,6	7,0	90,4	1,1	123,2	7,1	0,8	0,3	15,7	0,6
11,5	12	0,5	203,4	6,9	92,2	1,1	111,2	7,0			11,8	0,5
12	12,5	0,5	173,9	11,2	92,3	1,9	81,6	11,4			8,4	0,8
12,5	13	0,5	172,6	11,1	78,8	1,8	93,8	11,2	1,3	0,5	9,4	0,8
13	13,8	0,8	182,9	6,5	84,1	1,1	98,8	6,6			10,9	0,5
13,8	14,4	0,6	147,8	10,1	83,8	1,8	64,0	10,2			10,4	0,8
14,4	15,2	0,8	155,6	10,6	73,7	1,7	81,9	10,7	1,8	0,5	10,0	0,8
15,7	16,7	1	120,6	9,8	82,1	1,8	38,5	10,0			5,4	0,6
16,6	17,4	0,8	134,3	10,1	76,9	1,8	57,3	10,3			4,1	0,6
17,25	17,75	0,5	104,9	5,3	74,1	1,0	30,8	5,4			2,6	0,3
17,85	18,55	0,7	111,8	10,3	78,6	1,8	33,2	10,5			3,3	0,6
18,4	18,8	0,4	104,9	9,1	79,6	1,7	25,3	9,3			5,0	0,6

Table S7: Downcore radionuclide activities measured in the Eychauda Lake (EYC23) sediment core. Activities are expressed in mBq g^{-1}.

top (cm)	bottom (cm)	thickness (cm)	210Pb (mBq/g)	Err 210Pb (mBq/g)	226Ra (mBq/g)	Err 226 Ra (mBq/g)	210Pb ex (mBq/g)	err Pb exc (mBq/g)	241Am (mBq/g)	Err 241Am (mBq/g)	137Cs (mBq/g)	err 137 Cs (mBq/g)
0	0,8	0,8	579,1	15,0	63,6	1,4	515,6	15,1			64,1	1,6
0,8	1,6	0,8	474,3	12,9	64,7	1,3	409,6	13,0	1,7	0,5	72,9	1,6
1,6	2,4	0,8	408,1	13,0	74,6	1,5	333,5	13,1	1,0	0,5	75,2	1,7
2,4	3,5	1,1	489,9	12,2	72,6	1,3	417,3	12,3	1,6	0,4	77,7	1,5
3,5	4,3	0,8	384,2	11,0	61,5	1,2	322,7	11,0	0,9	0,4	67,8	1,4
4,3	5,2	0,9	504,9	13,7	81,4	1,5	423,6	13,8	1,2	0,5	111,2	1,9
5,2	6	0,8	428,1	12,0	70,4	1,3	357,6	12,0	0,9	0,4	122,4	1,9
6	6,9	0,9										
6,9	7,9	1	369,7	11,9	77,8	1,4	291,9	12,0	2,3	0,5	117,6	2,0
7,9	8,8	0,9	319,4	11,3	94,2	1,6	225,1	11,4	2,2	0,5	130,1	2,1
8,8	9,6	0,8	291,6	10,5	82,0	1,4	209,5	10,6	1,9	0,5	144,6	2,1
9,6	10,4	0,8										
10,4	11,4	1	335,1	11,0	90,2	1,5	245,0	11,1	2,6	0,5	253,8	2,7
11,4	12,3	0,9	287,6	11,1	85,2	0,3	202,5	11,1	2,4	0,5	361,7	3,4
12,3	14,6	2,3	293,0	10,8	100,5	1,6	192,5	10,9	3,0	0,5	242,5	2,7
14,6	17,4	2,8	283,8	11,0	95,5	0,3	188,3	11,0	2,1	0,5	137,2	2,2
17,4	18	0,6	267,5	10,2	90,0	1,5	177,5	10,3	3,1	0,5	166,0	2,2
18	18,5	0,5	253,7	10,2	93,3	1,5	160,3	10,3	3,9	0,5	202,7	2,5
18,5	19,3	0,8	210,7	9,2	85,2	1,4	125,4	9,3	5,5	0,5	212,8	2,5
19,3	20,2	0,9	183,3	9,0	85,3	0,3	98,0	9,0	4,3	0,5	167,7	2,3
20,2	20,9	0,7	182,7	8,9	86,9	1,5	95,9	9,0	2,4	0,4	73,1	1,5
20,9	22	1,1	175,8	4,9	86,7	0,1	89,1	4,9	1,7	0,2	57,9	0,8
22	23	1	154,3	8,4	75,9	1,4	78,4	8,5	1,5	0,4	23,8	0,9
23	23,7	0,7	139,3	5,6	77,4	1,0	61,9	5,7	0,9	0,3	27,2	0,7
23,7	24,3	0,6	136,6	5,3	74,6	0,9	62,0	5,4	1,1	0,3	14,4	0,5
24,3	28	3,7	112,3	6,7	74,8	0,2	37,5	6,7				
28	29,5	1,5	121,6	7,0	80,9	1,4	40,7	7,1				
29,5	30,5	1	110,4	4,2	80,1	0,8	30,3	4,3				
30,5	31,5	1	118,0	7,1	88,5	1,4	29,5	7,2				
31,5	32,4	0,9										
32,4	33,5	1,1	94,9	6,5	82,3	1,3	12,6	6,6				
18,4	18,8	0,4	104,9	9,1	79,6	1,7	25,3	9,3			5,0	0,6

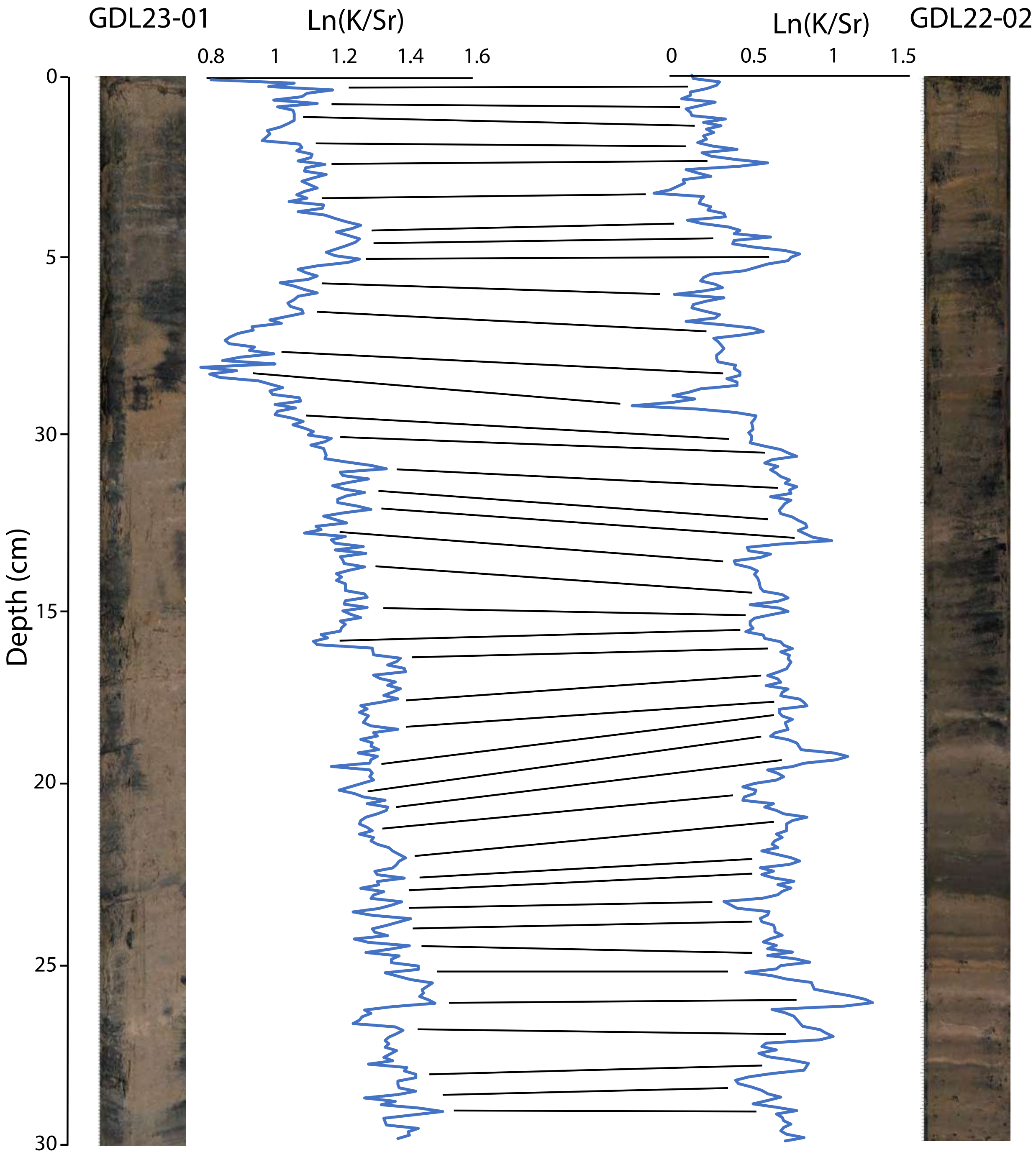

Figure S1: Core to core correlation based on K/Sr geochemical ratio in Grand Lake. On the left GDL23-01 on right GDL22-01.

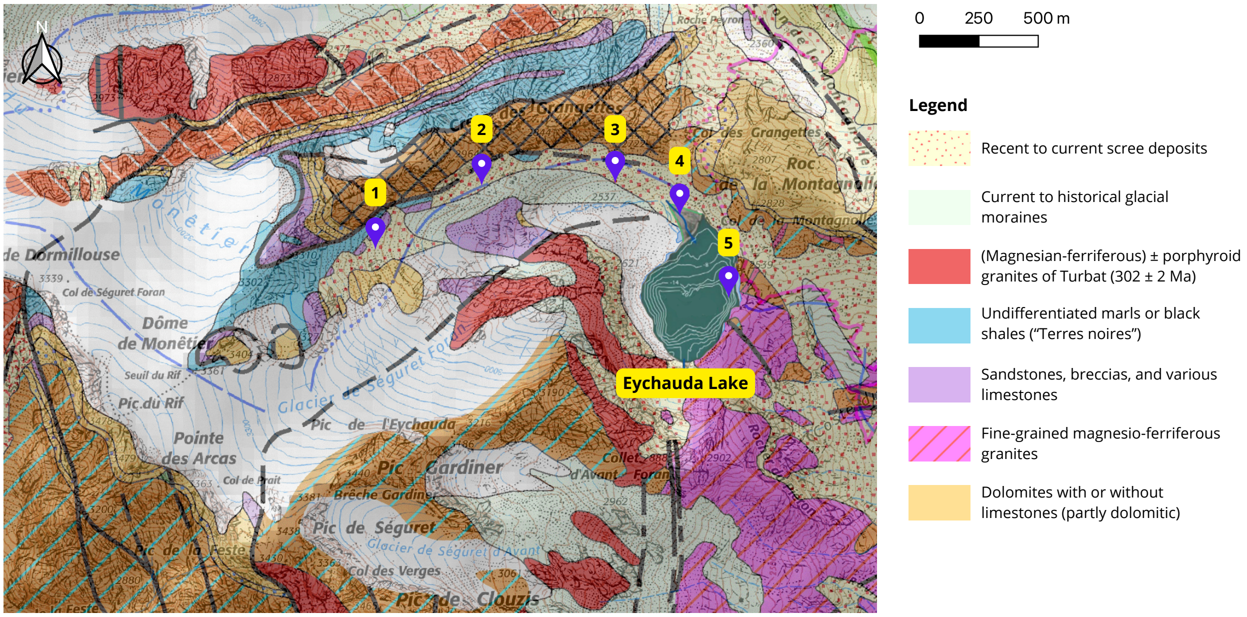

Figure S2: Geology of the Eychauda Lake(EYC) catchment (interactive map on the BRGM website), with Lake bathymetry and the locations of five soil/vegetation sampling points in the watershed

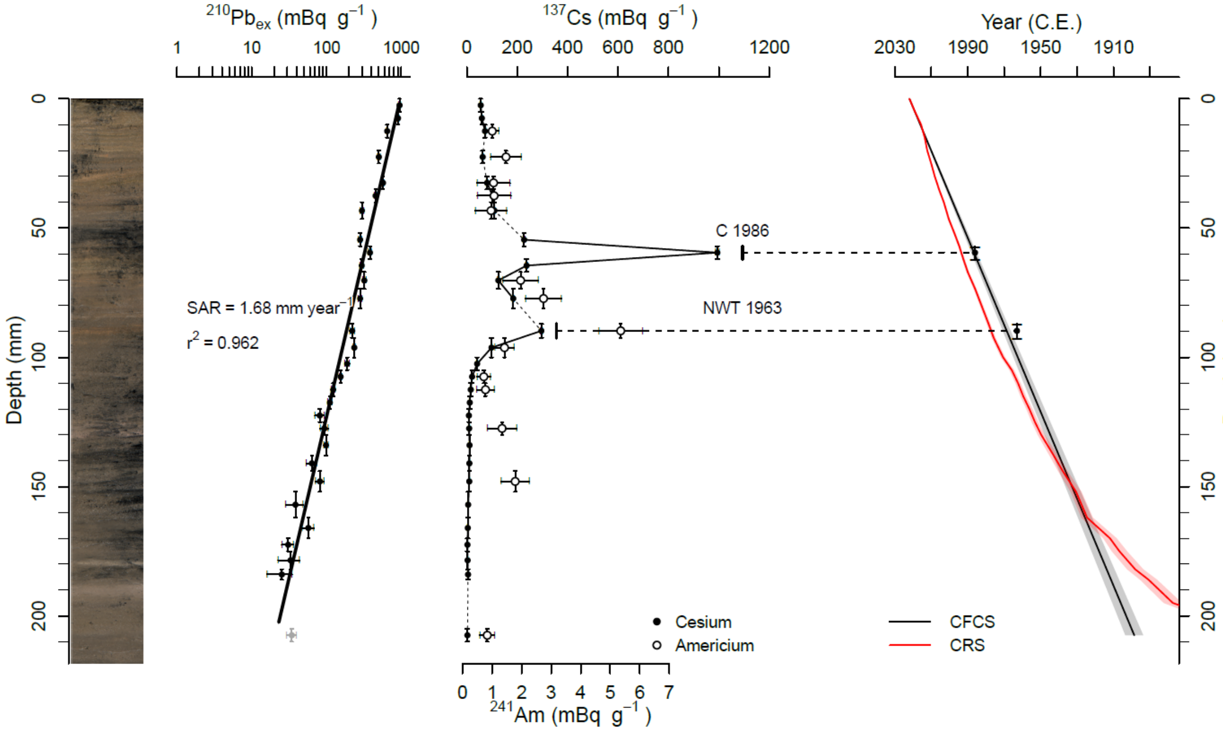

Figure S3: Vertical profiles of radionuclide activity in the GDL22 sediment core (Grand Lake), used for age-depth modeling. The excess ^{210}Pb activity (mBq g^{-1}) follows an exponential decay, with a strong linear regression fit (r^2 = 0.962), indicating a mean sediment accumulation rate (SAR) of 1.68 mm·yr^{-1}. Distinct peaks in ^{137}Cs and ^{241}Am correspond to known historical events, the 1963 Northern Hemisphere nuclear weapons testing maximum (NWT) and the 1986 Chernobyl accident, providing chronological validation.

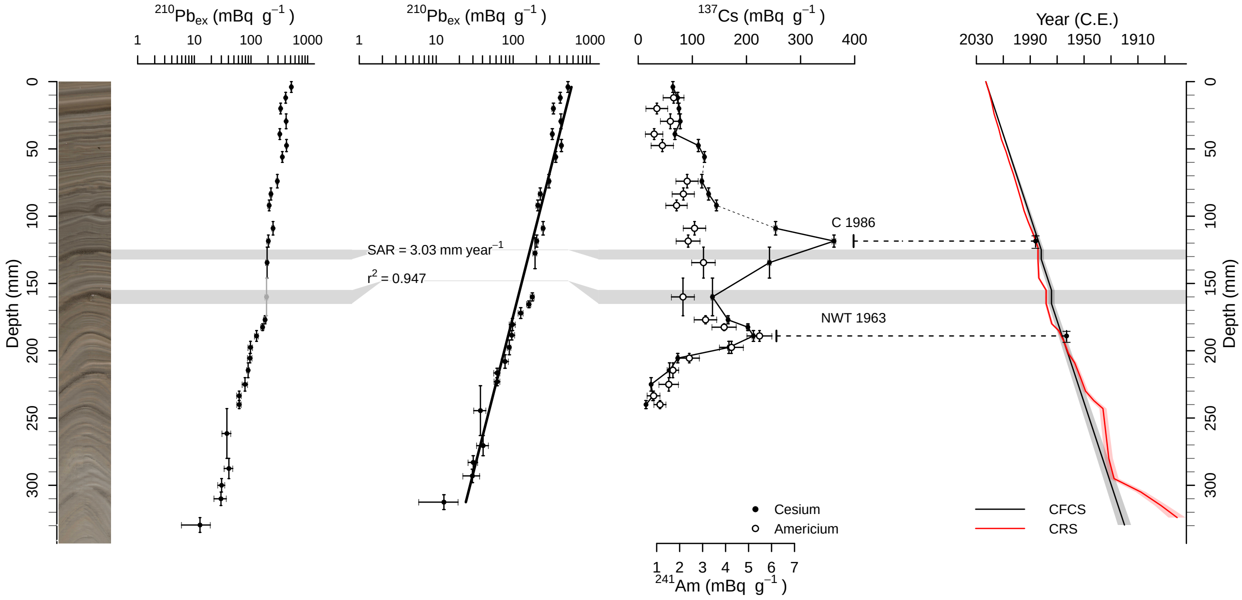

Figure S4: Vertical profiles of radionuclide activity in the EYC23 sediment core (Eychauda Lake), used for age-depth modeling. The excess ^{210}Pb activity (mBq·g^{-1}) follows an exponential decay, with a strong linear regression fit ($r^2 = 0.947$), indicating a mean sediment accumulation rate (SAR) of 3.03 mm·yr^{-1}. Distinct peaks in ^{137}Cs and ^{241}Am correspond to known historical events, the 1963 Northern Hemisphere nuclear weapons testing maximum (NWT) and the 1986 Chernobyl accident, providing chronological validation.

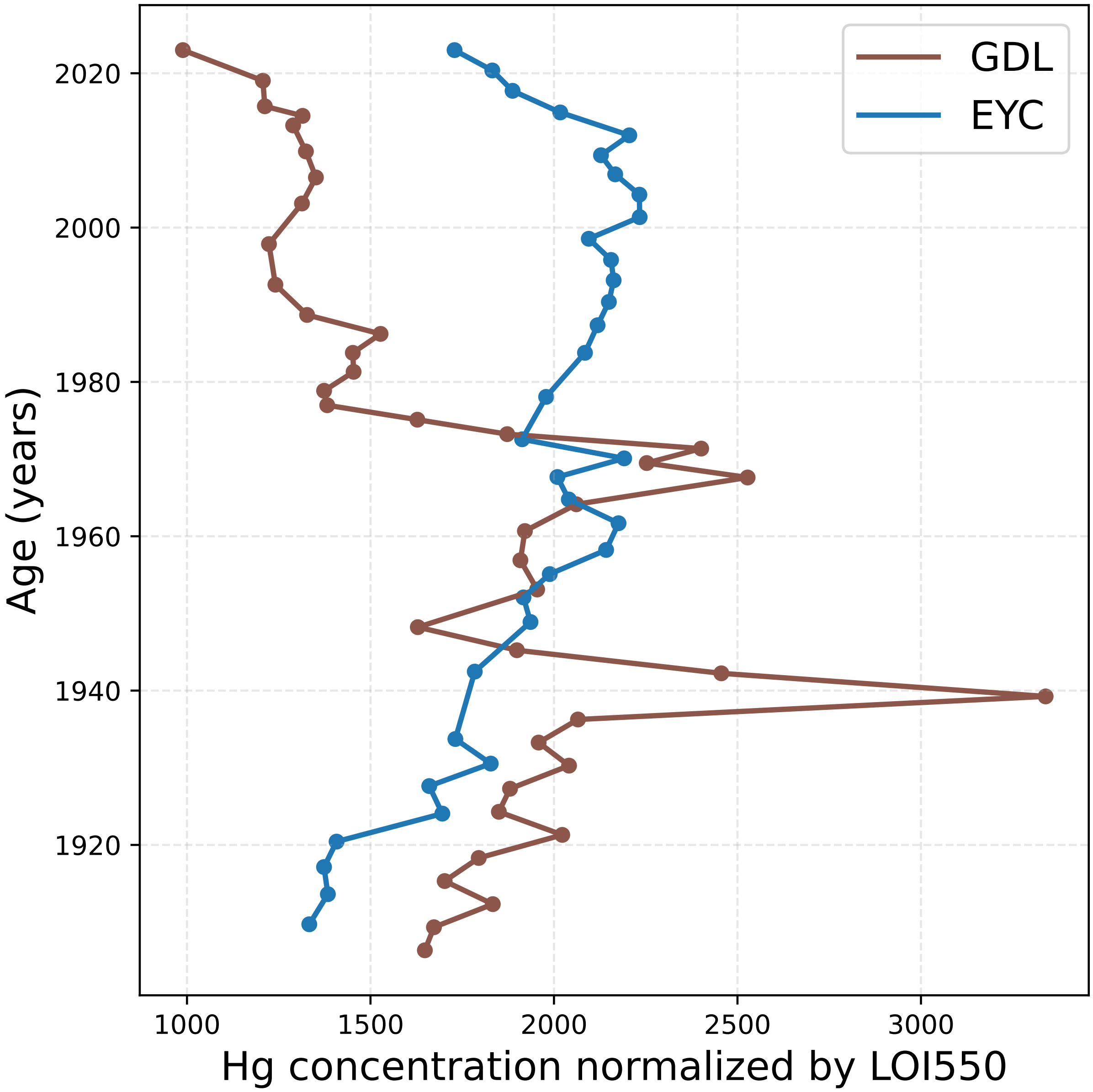

Figure S5: Mercury concentrations normalized to loss-on-ignition at 550°C (LOI_{550}) for the non-glacier-fed Grand Lake (GDL, in brown) and the glacier-fed Eychauda Lake (EYC, in blue) plotted against sediment age (y-axis). Normalization to LOI_{550} accounts for variations in sediment organic matter content and highlights the post-1970 divergence between the two sites.

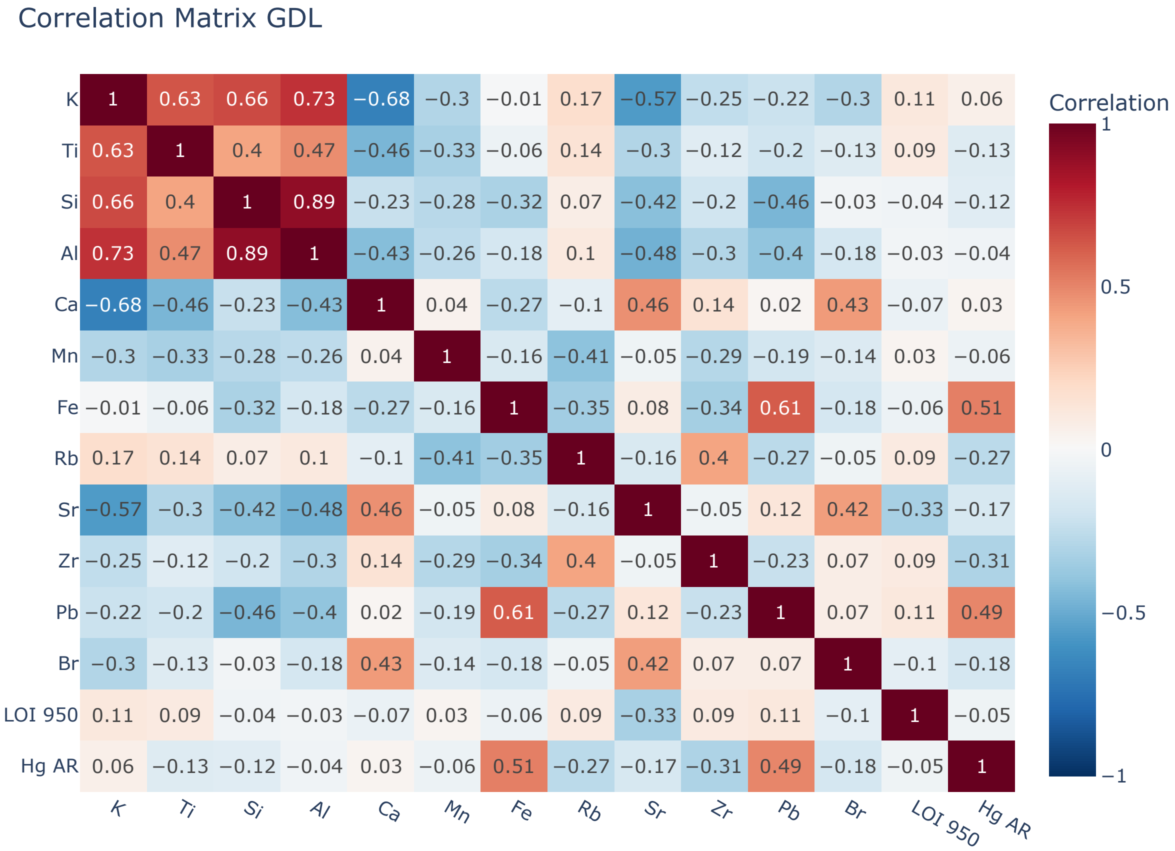

Figure S6: Correlation matrix of 12 elements derived from X-ray fluorescence (XRF) analysis, Loss on Ignition at 950°C (LOI 950), and Hg accumulation rate (AR) for the Grand Lake (GDL) sediment core.

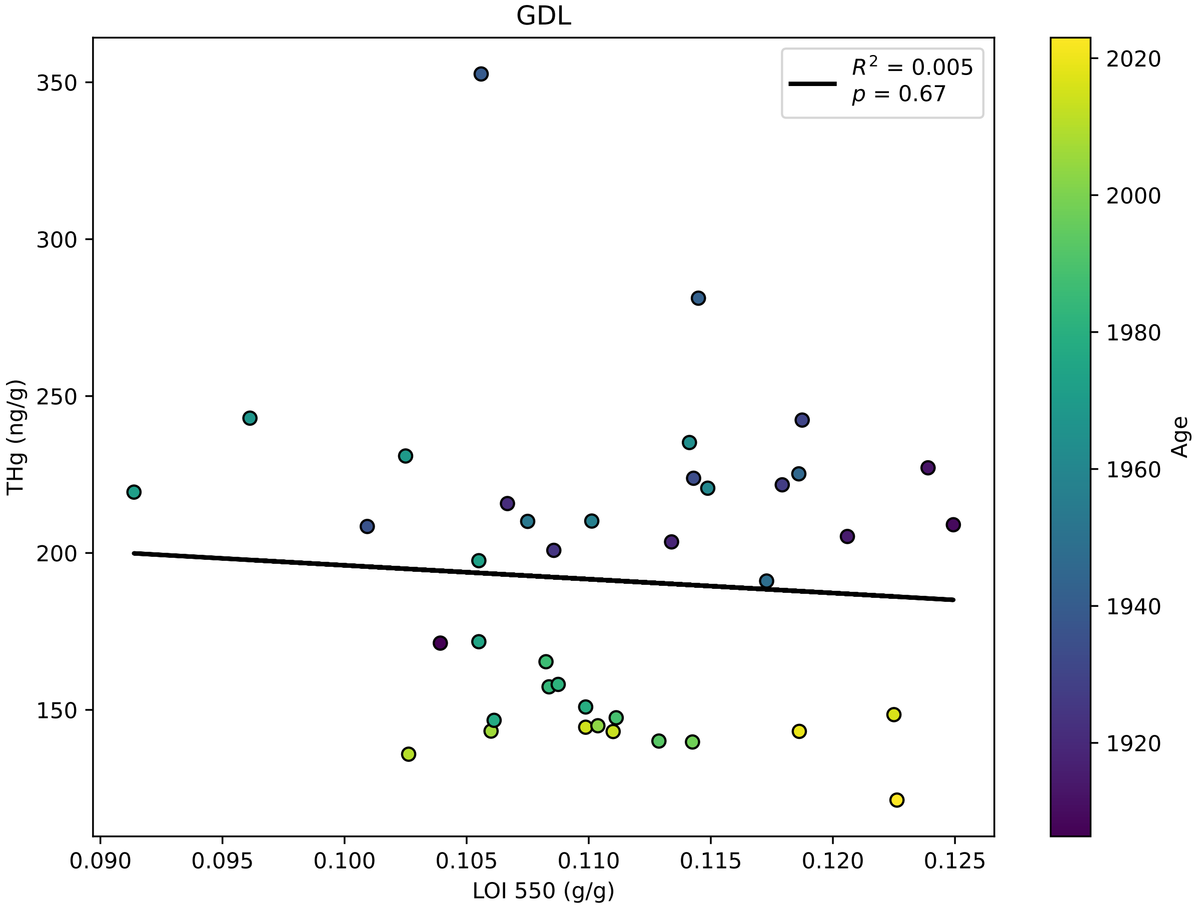

Figure S7: Total Hg (THg) concentration and loss of ignition at 550°C (LOI 550) linear regression for the non-glacier-fed Grand Lake (GDL). LOI 550 is a proxy of organic matter content.

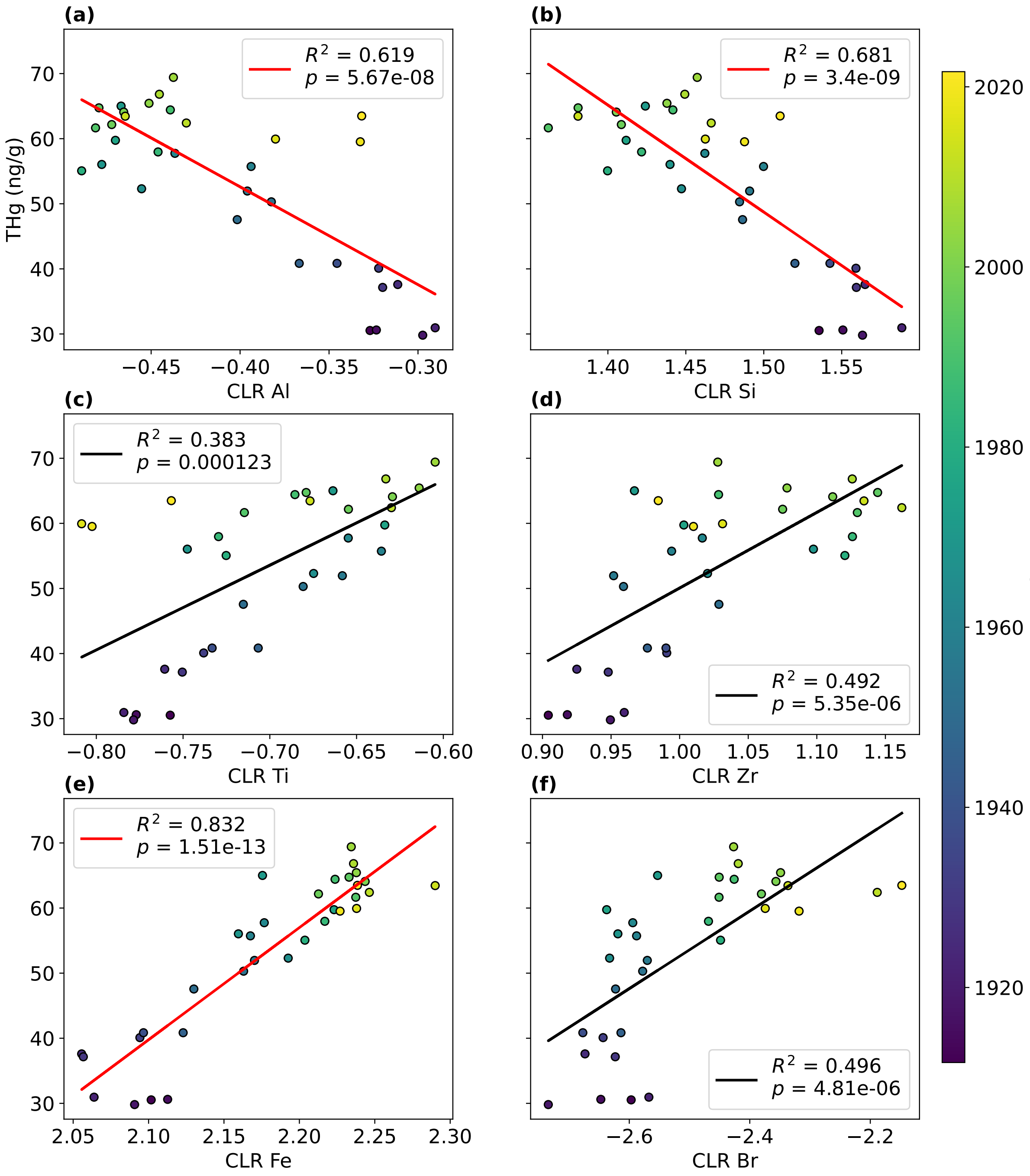

Figure S8: Linear regressions between Hg concentrations and centered log ratio (CLR) values of selected erosion proxy elements. The coefficient of determination (R^2) and p-value are indicated for each correlation. Statistically significant correlations ($R^2 > 0.6$) are highlighted in red. The color scale indicates sample age.

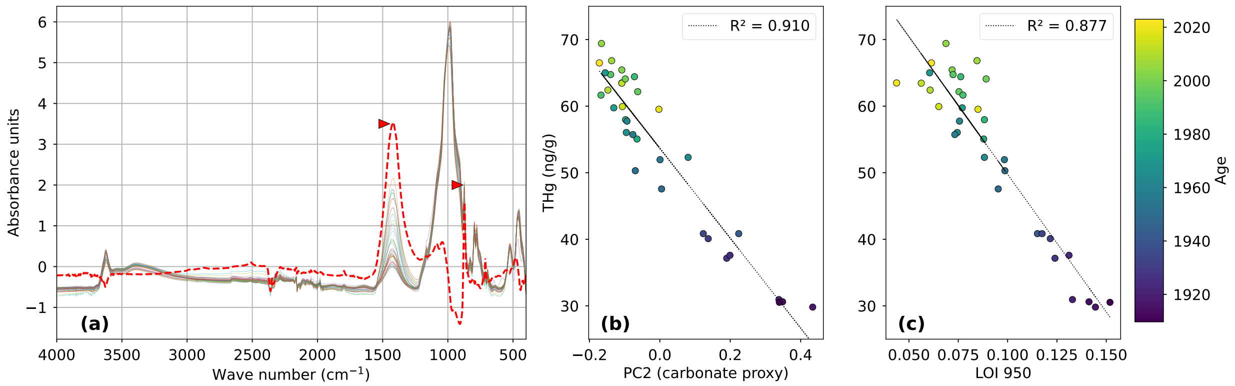

Figure S9: (a) FTIR-ATR spectra of sediment samples from the glacial-fed lake, with the red dashed line indicating the scores of Principal Component 2 (PC2). Carbonate absorption bands at 1460 cm^{-1} (C-O stretching) and 880 cm^{-1} (O-C-O bending) are highlithed with red arrows, where PC2 reached its maximum, indicating its association with carbonate-rich phases.[10] The peak at 1000 cm^{-1} corresponds to Si–O–Si stretching vibrations.[11] (b) Linear regression between Hg accumulation rate (AR) and the PC2 loading values. (c) Linear regression between Hg AR and loss-on-ignition at 950°C (LOI 950), used as a proxy of carbonate content in the sediment.

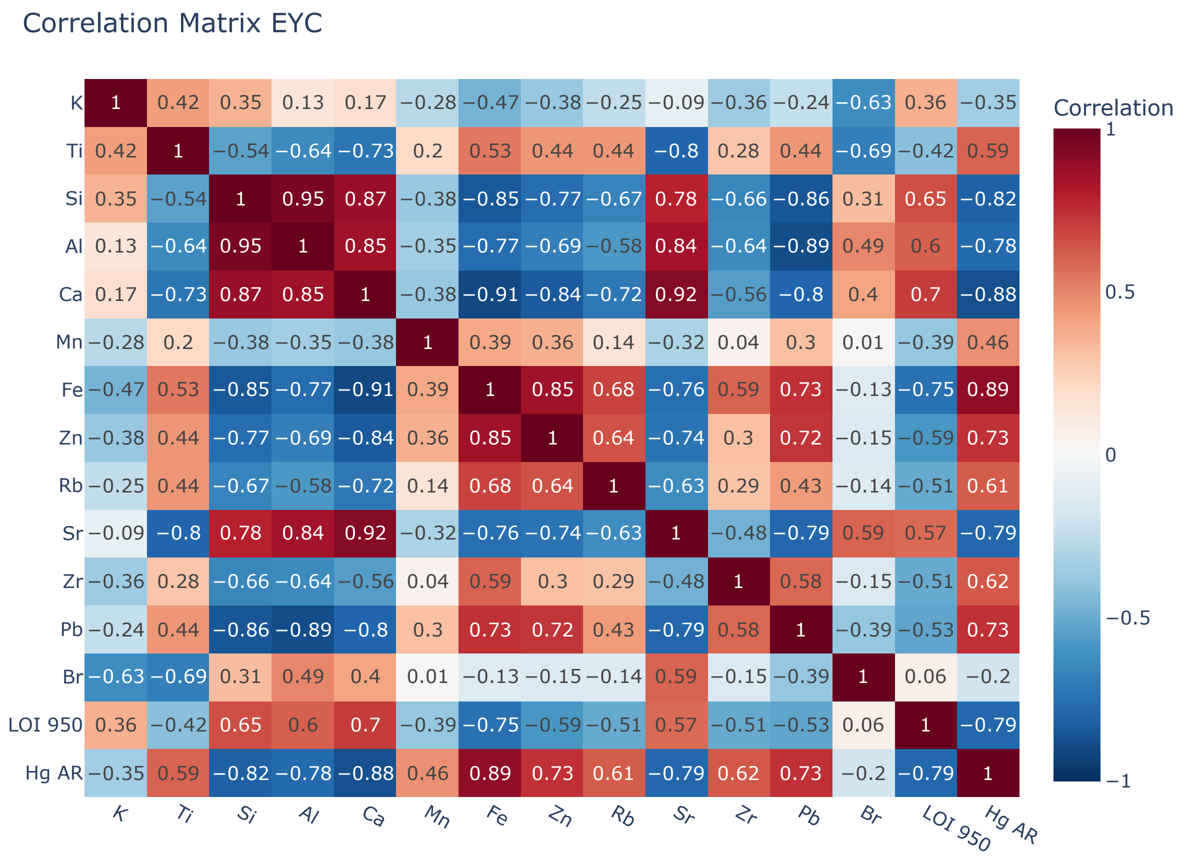

Figure S10: Correlation matrix of 12 elements derived from X-ray fluorescence (XRF) analysis, Loss on Ignition at 950°C (LOI 950), and Hg AR for the Eychauda (EYC) sediment core.

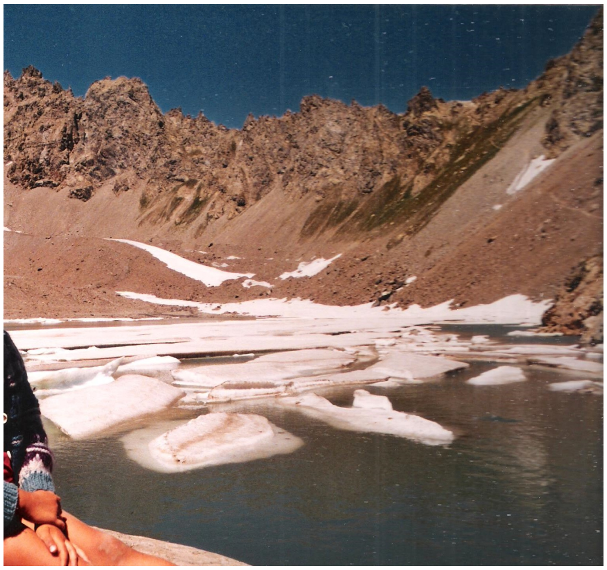

(a) *Eychauda Lake (1984)*

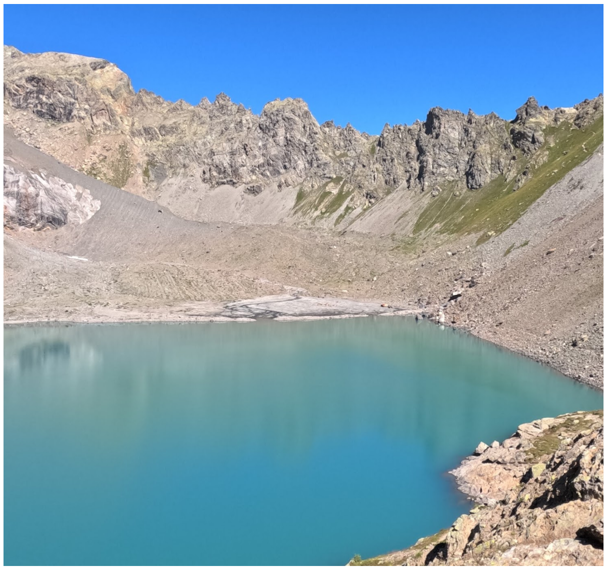

(b) *Eychauda Lake (2025)*

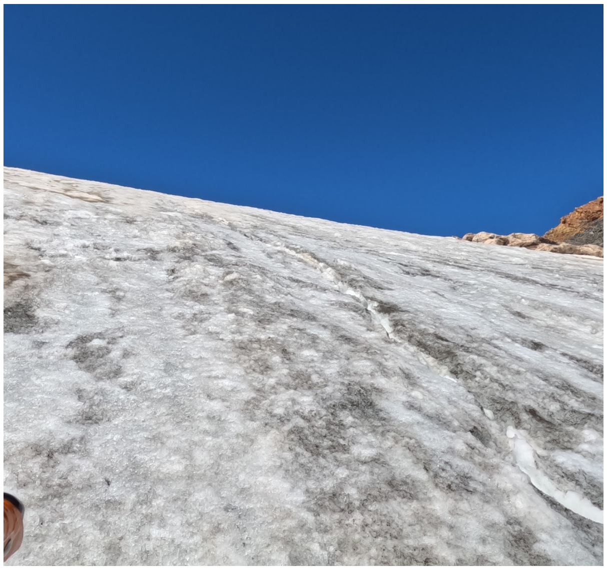

(c) *Ablation zone with particulate-rich ice (2025)*

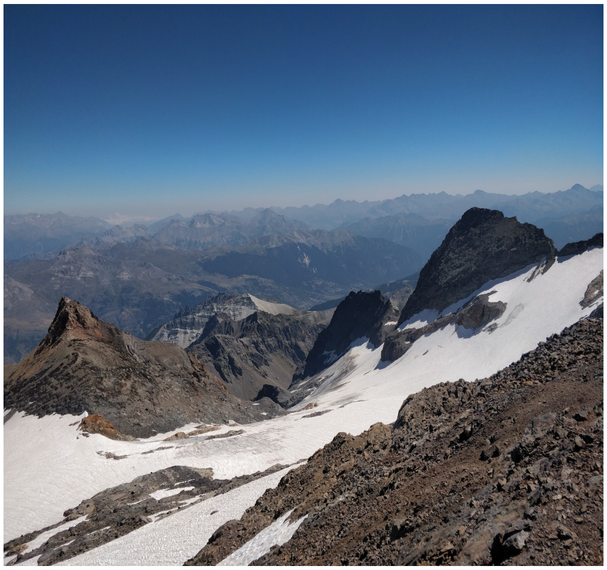

(d) *Séguret Foran Glacier (2025)*

Figure S11: Photographic documentation of the Eychauda watershed and the Séguret Foran Glacier. (a) Historical view of Eychauda Lake in 1984, compared with (b) its appearance in 2025 after major glacial retreat. (c) Ablation zone ice containing particulate-rich cryoconite deposits (2025). (d) Current view of the nearly vanished Séguret Foran Glacier (2025).

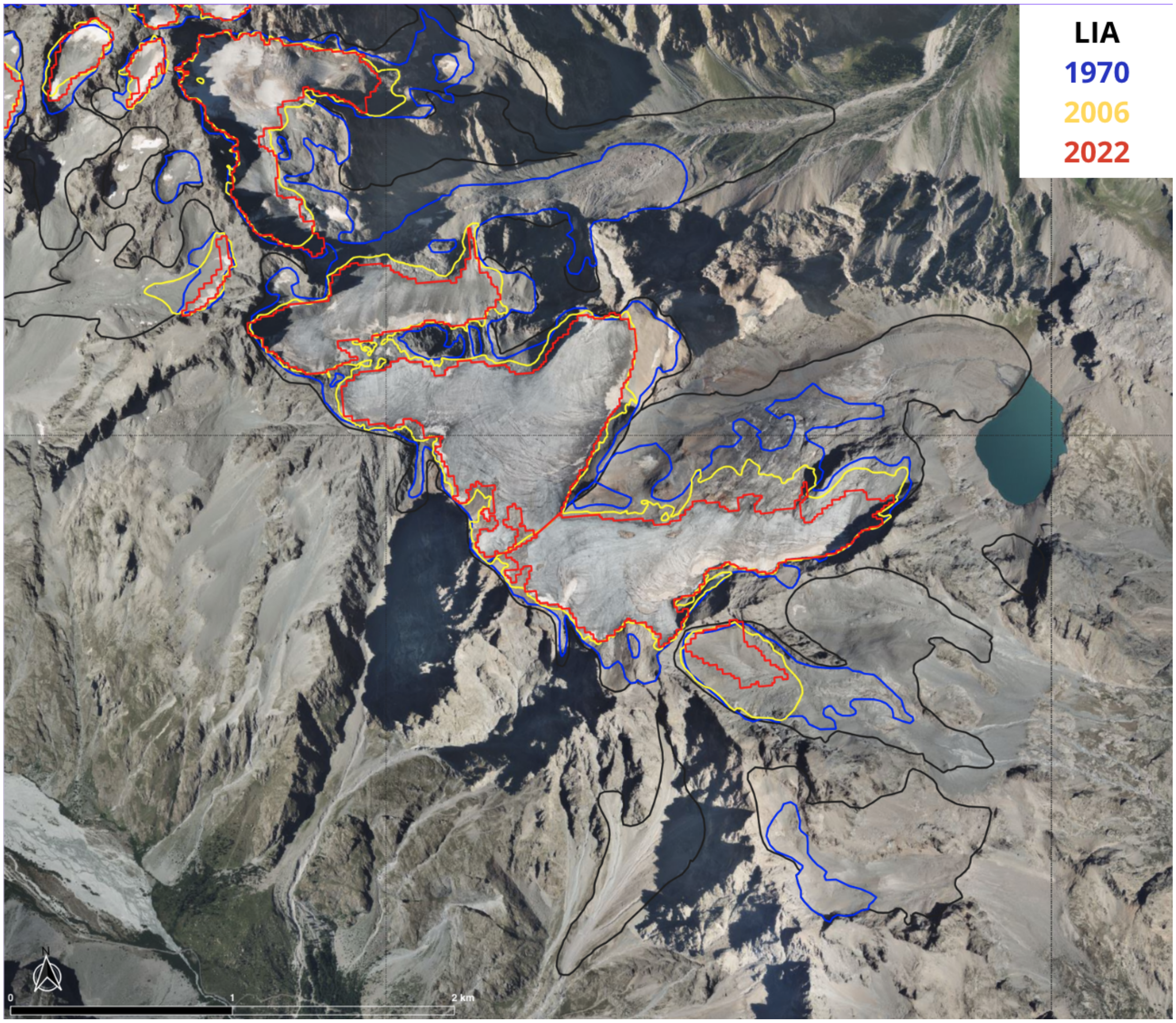

Figure S12: Map of the Eychauda Lake catchment showing reconstructed extents of the Séguret Foran Glacier from the Little Ice Age (c. 1840–1850) to 2022. Glacier outlines correspond to: LIA (black line), 1970 (blue), 2006 (yellow) and 2022 (red). Glacioclim/Copernicus satellite imagery

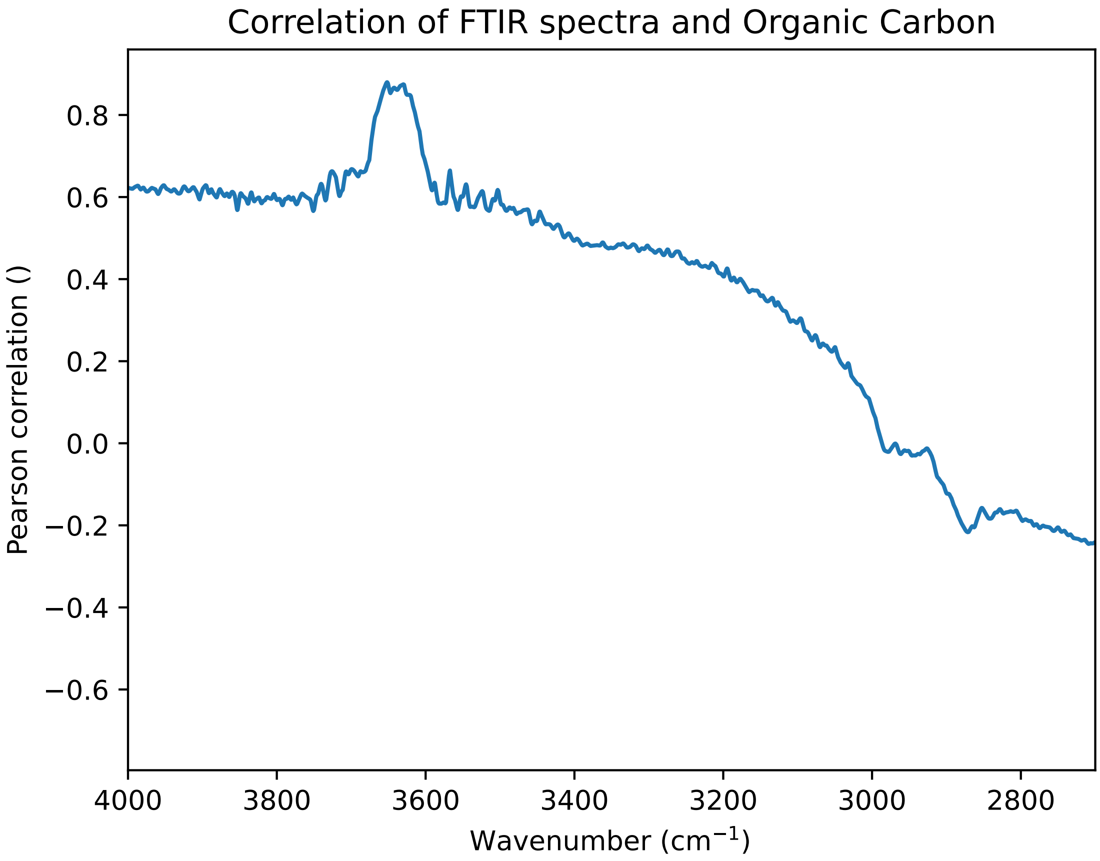

Figure S13: Pearson correlation between FTIR spectra and total organic carbon (TOC) content in Eychauda Lake sediments. A strong positive correlation is observed in the wavenumber range 3690–3620 cm^{-1}, consistent with clay mineral vibrations, indicating that clay-rich phases are important carriers of organic carbon.

References

(1) Bolibar, J.; Rabatel, A.; Gouttevin, I.; Galiez, C.; Condom, T.; Sauquet, E. Deep learning applied to glacier evolution modelling. *The Cryosphere* **2020**, *14*, 565–584.

(2) Gardent, M.; Rabatel, A.; Dedieu, J.-P.; Deline, P. Multitemporal glacier inventory of the French Alps from the late 1960s to the late 2000s. *Global and Planetary Change* **2014**, *120*, 24–37.

(3) Paul, F.; Rastner, P.; Azzoni, R. S.; Diolaiuti, G.; Fugazza, D.; Le Bris, R.; Nemec, J.; Rabatel, A.; Ramusovic, M.; Schwaizer, G.; Smiraglia, C. Glacier shrinkage in the Alps continues unabated as revealed by a new glacier inventory from Sentinel-2. *Earth System Science Data* **2020**, *12*, 1805–1821.

(4) Cooke, C. A.; Martínez-Cortizas, A.; Bindler, R.; Sexauer Gustin, M. Environmental archives of atmospheric Hg deposition – A review. *Science of The Total Environment* **2020**, *709*, 134800.

(5) Paudyal, R.; Kang, S.; Guo, J.; Tripathee, L.; Sharma, C. M.; Huang, J.; Li, Y.; Yan, F.; Wang, K.; Chen, J.; Qin, X.; Sillanpaa, M. Mercury sources and physicochemical characteristics in ice, snow, and meltwater of the Laohugou Glacier Basin, China. *Environmental Science and Pollution Research* **2021**, *28*, 51530–51543.

(6) Huang, J.; Kang, S.; Ma, M.; Guo, J.; Cong, Z.; Dong, Z.; Yin, R.; Xu, J.; Tripathee, L.; Ram, K.; Wang, F. Accumulation of Atmospheric Mercury in Glacier Cryoconite over Western China. *Environmental Science & Technology* **2019**, *53*, 6632–6639, PMID: 31117527.

(7) Baccolo, G.; Di Mauro, B.; Massabò, D.; Clemenza, M.; Nastasi, M.; Delmonte, B.; Prata, M.; Prati, P.; Previtali, E.; Maggi, V. Cryoconite as a temporary sink for anthropogenic species stored in glaciers. *Scientific Reports* **2017**, *7*, 9623, Publisher: Nature Publishing Group.

(8) Takeuchi, N.; Kohshima, S.; Seko, K. Structure, Formation, and Darkening Process of Albedo-reducing Material (Cryoconite) on a Himalayan Glacier: A Granular Algal Mat Growing on the Glacier. *Arctic, Antarctic, and Alpine Research* **2001**, *33*, 115–122.

(9) Takeuchi, N.; Li, Z. Characteristics of Surface Dust on Ürümqi Glacier No. 1 in the Tien Shan Mountains, China. *Arctic, Antarctic, and Alpine Research* **2008**, *40*, 744–750.

(10) Mecozzi, M.; Pietrantonio, E.; Amici, M.; Romanelli, G. Determination of carbonate in marine solid samples by FTIR-ATR spectroscopy. *Analyst* **2001**, *126*, 144–146.

(11) Ellerbrock, R.; Stein, M.; Schaller, J. Comparing amorphous silica, short-range-ordered silicates and silicic acid species by FTIR. *Scientific Reports* **2022**, *12*, 11708, Publisher: Nature Publishing Group.
